# Coercivity‐Size Map of Magnetic Nanoflowers: Spin Disorder Tunes the Vortex Reversal Mechanism and Tailors the Hyperthermia Sweet Spot

**DOI:** 10.1002/smsc.202500490

**Published:** 2025-11-05

**Authors:** Elizabeth M. Jefremovas, Lisa Calus, Jonathan Leliaert

**Affiliations:** ^1^ Department of Physics and Materials Science University of Luxembourg 162A Avenue de la Faiencerie L‐1511 Luxembourg Luxembourg; ^2^ Institute for Advanced Studies University of Luxembourg Campus Belval L‐4365 Esch‐sur‐Alzette Luxembourg; ^3^ DyNaMat, Department of Solid State Sciences Ghent University 9000 Ghent Belgium

**Keywords:** iron oxide nanoparticles, magnetic particle hyperthermia, micromagnetic simulations, nanoflowers, nanomagnetism, spin disorder

## Abstract

Iron‐oxide nanoflowers (NFs) are one of the most efficient nanoheaters for magnetic hyperthermia therapy. However, the physics underlying the dynamic response of realistic nanoparticles, containing disorder, beyond the single‐domain limit remains poorly understood. Using large‐scale micromagnetic simulations, the magnetization of biocompatible iron‐oxide NFs (*d* = 10–400 nm) has been mapped, connecting their microstructure to their macroscopic magnetic response. Above the single‐domain regime (*d* > 50 nm), the magnetization folds into a vortex state, within which the coercivity reaches a secondary maximum, not present for nondisordered nanoparticles. The dynamics of the vortex shows two distinct reversal modes: 1) a core‐dominated one, with an increasing coercivity with *d*; 2) a flux‐closure‐domains dominated reversal mode, with a decreasing coercivity‐size dependence. The coercivity maximum is located at the transition between both reversal modes and results from the combination of grain anisotropy and grain‐boundary pinning. The results provide the first description of spin textures in iron oxide NFs beyond the macrospin framework, revealing how particles with identical static magnetization exhibit fundamentally distinct dynamics, which result in different macroscopic behavior. By adjusting the grain size, the coercivity “sweet spot” can be tailored, offering a practical route to next‐generation, high‐efficiency nanoheaters.

## Graphical abstract

Large‐scale micromagnetic simulations show how disorder and grain boundaries control magnetization dynamics in iron‐oxide nanoflowers beyond the single‐domain regime. Two competing vortex‐reversal modes emerge, producing a coercivity maximum at their transition. Linking microstructure to macroscopic response, this work demonstrates how tuning intraparticle disorder via grain size manipulation enables more efficient and controllable magnetic hyperthermia nanoheaters.

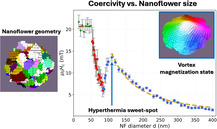

## 1 Introduction

Magnetic nanoparticles (MNPs) are at the forefront of a new era in personalized medicine, hosting potential in both diagnostic and therapeutic applications.^[^ ^1^, ^2^, ^3^, ^4^, ^5^, ^6^ ^]^ Their highly tunable magnetic response and size enables targeted approaches, such as magnetic hyperthermia treatment (MHT), where local heating selectively disrupts malignant cells, providing a promising complement to conventional cancer therapies.^[^ ^7^, ^8^, ^9^, ^10^, ^11^ ^]^ Among MNPs, iron oxide nanoparticles (IONPs) are especially well suited owing to their excellent biocompatibility and magnetic tunability, which together enable the design of personalized treatment strategies aligned with the requirements of next‐generation nanomedicine.^[^ ^12^, ^13^, ^14^, ^15^, ^16^ ^]^

A key challenge in MHT is to maximize the heating power of IONPs, achieving effective thermal responses with minimal nanoparticle dosage. Several strategies have been explored to achieve this aim, such as tuning the composition, size, shape, and degree of disorder, as well as engineering supraparticle arrangements like chains.^[^ ^6^, ^17^, ^18^, ^19^, ^20^, ^21^, ^22^ ^]^ Nonetheless, these efforts have often been carried out without a full understanding at microscopic level of how these factors influence magnetic heating performance, thereby limiting their predictive power. In particular, both the spin texture, i.e., the spatial arrangement of magnetic moments inside the nanoparticle, and the dynamic response of that texture to externally applied magnetic fields are essential for heat dissipation, yet their roles remain poorly understood. Bridging the nanoparticle microstructure to its macroscopic magnetic properties, such as coercivity, is the bottle‐neck for the rational design of next‐generation hyperthermia agents.

One particular spin texture highly relevant for biomedical applications is the vortex state.^[^ ^23^, ^24^ ^]^ This inhomogeneous spin texture minimizes stray magnetic fields, suppressing interparticle dipolar interactions, thereby preventing undesired magnetic aggregation.^[^ ^25^, ^26^ ^]^ At the same time, the vortex core retains a net magnetization, which can contribute to heating or serve as a vector for drug delivery.^[^ ^23^, ^24^, ^27^, ^28^ ^]^ Theoretically, vortex states are predicted in fine ferromagnetic nanoparticles exceeding the single‐domain size limit,^[^ ^29^, ^30^, ^31^, ^32^ ^]^ with the critical size depending on material properties.^[^ ^33^, ^34^, ^35^, ^36^, ^37^, ^38^, ^39^, ^40^ ^]^ Experimentally, vortex states have been observed in IONPs of different morphologies, including disks, ellipsoids, spheres, or cubes.^[^ ^23^, ^28^, ^41^, ^42^, ^43^ ^]^ More recently, vortex states were detected in multicore nanostructures called nanoflowers (NFs).^[^ ^44^ ^]^ NFs perform exceptionally in MHT,^[^ ^19^, ^45^, ^46^, ^47^ ^]^ and their synthesis is already scaled to commercial production,^[^ ^21^, ^48^, ^49^ ^]^ making them strong candidates for clinical translation in magnetic hyperthermia.^[^ ^50^ ^]^

Despite growing interest in vortex configurations in nanoparticles, the theoretical interpretation of experimental observations remains incomplete. Detecting clearly and unambiguously which features the models should capture to accurately explain the magnetization in complex morphologies, like NFs, remains challenging. Most theoretical studies employ idealized models that include only demagnetizing and exchange interactions (e.g., ref. [29]). Experiments, however, reveal that anisotropy and spin disorder, both intrinsic to nanostructured systems, play key roles in stabilizing nonuniform spin textures.^[^ ^46^, ^51^, ^52^, ^53^ ^]^ More precisely, spin disorder, originating at magnetic imperfections, and always present in nanoparticles as a consequence of surface effects (lower symmetry, reduced coordination), promotes inhomogeneous magnetic patterns, yet its influence on magnetization dynamics remains unclear.^[^ ^53^, ^54^, ^55^, ^56^ ^]^ Elucidating the physics of intraparticle spin disorder is crucial to exploit its full application potential, as theory predicts that magnetic defects could raise the heating efficiency by up to an order of magnitude compared with defect‐free nanoparticles.^[^ ^22^ ^]^

In this work, we go beyond the macrospin framework and perform a full micromagnetic numerical study to resolve the inhomogeneous spin texture of magnetic NFs, revealing how intraparticle spin disorder governs their magnetization reversal. By connecting local magnetic inhomogeneities to macroscopic coercivity, we demonstrate that tuning the degree of internal disorder enables control over the threshold NF size required to stabilize vortex states, as well as the NF size at which coercivity is maximized. Our findings bridge the nanoparticle microstructure, determined by the spin texture, and heating performance, represented by the coercivity, offering unique and fundamental insights to tailor the coercivity “sweet spot” for optimized hyperthermia performance.

The article is structured as follows: in Section 2.1 we present the full coercivity versus NF diameter map, from diameter *d* = 10 nm to *d* = 400 nm, identifying the secondary maximum of coercivity for the vortex regime. Section 2.2 details the magnetization reversal mechanisms of the vortex phase, revealing two distinct switching regimes determined by NF size. Finally, Section 2.3 elucidates how the grain size and other disorder details like the exchange coupling reduction at grain boundaries affect coercivity.

## 2 Results and Discussion

**Figure 1:**
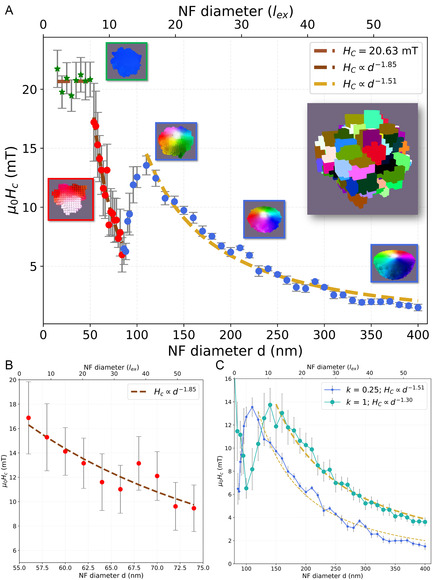
Coercivity versus size for NF‐like nanoparticles. A) Coercivity‐size map as a function of NF diameter for *k *= 0.25 and grain size = 15 nm. Beyond the single‐domain SW regime (green stars, *d *< 50 nm, single grain) there appears another single‐domain regime, but with a reversal not via coherent rotation (red dots, 50 nm < *d* < 75 nm). For even larger diameters, a vortex is stabilized within the whole range (blue dots). The insets show the micromagnetic structure (equatorial plane) at remanence ($\mu_{0}H_{0}=0T$) for *d *= 30, 70, 120, 200, and 400 nm. After reaching a maximum at *d* = 110 nm, the coercivity drops $\propto d^{-1.5}$. Top right inset includes the simulated grain microstructure of NF *d* = 100 nm, where the uniaxial anisotropy direction for each grain is represented in different colors. B) Zooms‐in on the single‐domain to vortex region, where the coercivity drops $\propto d^{-1.85}$ before the rise in the vortex regime. C) Comparison between the k=0.25 and k=1 cases. The onset of the vortex, together with the maximum of the coercivity (and thus, of the hyperthermia sweet spot), is shifted to larger values at k=1. Data shown as mean ± SEM across seeds. $\text{SEM}=\sigma /\sqrt{n}$; being the number of seeds n=20 per diameter *d*.

Our numerical study is designed to mirror the structure and behavior of maghemite NFs as realistically as possible. From a structural perspective, NFs present a nearly spherical shape, which preserves the advantages of single‐core spherical nanoparticles: almost isotropic coercivity and uniform heat release irrespective of the field orientation.^[^ ^57^ ^]^ Moreover, the rough surface and dense network of grain boundaries creates an intraparticle energy landscape rich in pinning sites, allowing spin disorder to influence the magnetization dynamics. We have included in **Figure** **1**A as an inset a representative image of our simulations, which reproduce such unique and distinct multicore morphology. As such, we have modeled the NF as a quasispherical cluster of crystalline grains, each with its own uniaxial anisotropy axis $K_{u}$ (indicated by different colors). We have turned on the parameter of spin disorder by lowering the exchange stiffness *A* at the grain boundaries with a scaling factor *k* (0≤k≤1), so the intragrain stiffness is *A* and intergrain stiffness becomes kA. This parameter lets us systematically probe how pinning affects the magnetization dynamics and, ultimately, heating performance.

Note that all simulations are performed in the absence of thermal fluctuations, i.e., at *T* = 0 K. This is imposed by computational costs and practicality. Introducing a stochastic thermal field term would increase dramatically the amount of computing time needed to reproduce such large‐scale study while yielding little to no additional insights in the vortex regime, which is the focus of our study. Note that thermal fluctuations would only affect the magnetization for nanoparticle sizes below the superparamagnetic limit. As shown below, for the present study of maghemite nanoparticles, the vortex regime lies above this limit, and as such, the influence of thermal fluctuations is minimal to the general conclusions of our findings.

### 2.1 Coercivity versus NF size

Figure 1 maps the evolution of the coercive field, $\mu_{0}H_{C}$, as a function of the NF diameter *d*, from 10 to 400 nm in steps of 10 nm. Note that reproducing such a wide range at the required precision in particle and grain size is beyond current experimental possibilities. As such, our numerical study provides the insights necessary to guide and interpret experimental measurements within the relevant size range. Diameters are plotted both in absolute units (nm, bottom axis) and normalized to the exchange length, $l_{\text{ex}}=\sqrt{2A/\mu_{0}M_{s}^{2}}$ (top axis), allowing material‐independent comparison and bridging to intrinsic magnetic length scales.

Unless otherwise specified, all simulations use a grain size of 15 nm and an intergrain coupling factor k=0.25, following experimental measurement data.^[^ ^19^, ^44^, ^45^, ^49^ ^]^ For comparison, a fully coupled case (k=1) is also included to isolate the effect of grain boundaries while preserving the NF geometry. Diameters from 10 to 400 nm are examined, fully covering the relevant experimental size range.^[^ ^18^, ^19^, ^44^, ^45^, ^47^, ^49^, ^58^, ^59^ ^]^

As commented in the previous section, our simulations are performed at *T* = 0 K, i.e., in the absence of thermal fluctuations. Such thermal fluctuations are only expected to influence the magnetization dynamics within the superparamagnetic regime, where they cause spontaneous magnetization switches. For spherical maghemite nanoparticles with uniaxial magnetocrystalline anisotropy, the superparamagnetic limit $d_{\text{SPM}}$ is estimated at ≈25 nm, estimated from the commonly accepted criterion for magnetic stability $\Delta E/(k_{B}T>25$).^[^ ^60^ ^]^ For the case of the present NFs, a lower bound of the energy barrier is expected, consequence of the random orientation of magnetocrystalline anisotropy axes in the individual crystallites forming the NF. This effective anisotropy is proportional to the number of grains, *N*, as $K_{\text{eff}}=\frac{K_{u}}{\sqrt{N}}$, from where the energy barrier can be estimated as $\Delta E=K_{u}\sqrt{V\;V_{g}}$, where *V* and $V_{g}$ represent the total NF volume and the grain volume. Given a grain size of 15 nm, the superparamagnetic limit is estimated at a diameter of 50 nm, which is below the vortex regime ($d_{c}=70\;\text{nm}$). Note that this estimate represents a lower limit, as effects such as pinning at grain boundaries due to reduced exchange coupling can further increase the energy barriers. Using the same expression, we estimate that our *d* = 100 nm‐sized NFs with 15 nm grain size would start to display thermal switching at a temperature value $T_{\text{SPM}}\;=\;\frac{K\;\sqrt{V\;V_{g}}}{25\;k_{B}}\approx 880K$, further confirming the negligible influence of thermal effects within the hyperthermia operating temperature, close to room temperature.^[^ ^8^, ^9^, ^61^ ^]^ Thereby, the physics underlying the emergence of the vortex and its dynamics, central to the results in this article, are not compromised by leaving thermal fluctuations out. At most they would impose a minor correction to the obtained coercivity values, but would leave their qualitative behavior unchanged.

The coercivity values obtained agree with the experimental data available in the literature, e.g., refs. [19, 44, 45, 62]. Representative complete hysteresis loops for 20 different random initial configurations per NF size are included in S2, Supporting Information. This Supporting Information section also includes a comparison between the energy losses per magnetic field cycle extracted from our simulations and the specific absorption rate (SAR) values reported from experiments. The reasonable agreement between simulations and experiments, accompanied by a discussion on the equivalence between both quantities, validates the use of coercivity as a proxy for the heat generation extracted from our simulations.

Furthermore, Figure 1A reveals three size regimes, each with a distinct, qualitatively different, coercivity trend.

In the smallest regime (*d* < 50 nm, green stars), the diameter lies below the single‐domain limit. Accordingly, we model every NF as a single grain. Note that simulations that explicitly resolve 15 nm grains are methodologically challenging because of the small and irregular number of grains per particle, at no additional value compared to modeling the NF as a single‐domain entity. To confirm that our approximation is adequate, we have included the results corresponding to the simulations performed with such grain size at this low *d* regime in Section S3, Supporting Information. There, Figure S2, Supporting Information, shows that even at the upper bound (*d *= 50 nm, ≈15 grains) the grain structure does not result in any deviations from the single‐domain and single‐grain behavior.

In our single grain simulations, the magnetization remains uniform throughout the entire hysteresis loop (see snapshot included in green at remanence), indicating that the reversal mechanism is a coherent rotation. Under these conditions the data can be interpreted using the Stoner–Wolhfarth (SW) model,^[^ ^63^ ^]^ where the coercive field remains constant and is given by $\mu_{0}H_{C}=0.48\frac{2K}{M_{s}}$, being the factor 0.48 included to account for the random distribution of the anisotropy axes.^[^ ^64^ ^]^ For our material parameters, this idealized model predicts a constant $\mu_{0}H_{C}=24\;\text{mT}$, which is slightly larger but in reasonable agreement with the numerical results of $H_{C}=20.6\;\text{mT}$.

The second regime spans between 50 nm < d < 75 nm (red dots in Figure 1A). Once the diameter exceeds the single‐domain threshold of $7.2l_{\text{ex}}$ ^[^ ^29^ ^]^ (51 nm for our material) reversals do no longer happen through a coherent rotation but rather through domain‐wall nucleation (see inset marked in red), which lowers the coercive field relative to the SW limit observed for smaller particles. Unlike the constant value of the coercivity within the SW regime, at this regime, the coercivity scales with size as $H_{C}\propto d^{-1.85}$. This change in the coercivity dependence is a direct consequence of the NF size exceeding the single‐domain regime. Accordingly, above d=50 nm, the internal grain structure of the NFs, with each grain having an independent random anisotropy easy axis, becomes dominant. The random anisotropy axes average the total anisotropy out to an effective anisotropy, $K_{\text{eff}}\propto 1/\sqrt{N}$, where *N* is the number of grains. Because a quasispherical NF contains $N\propto V\propto d^{3}$ grains, $H_{C}\propto K_{\text{eff}}\propto d^{-\frac{3}{2}}$. The fitting of our simulated results, included in Figure 1B, yields $H_{C}\propto d^{-1.85}$, indicating a reasonable agreement with this simplified model. We attribute the slightly steeper decline than the −1.5 power law expected for perfect spheres to disorder associated with the irregular edges of the NFs.

The third regime (blue symbols in Figure 1A) starts once the NF reaches a diameter large enough to host a vortex state at remanence (see blue insets). In our material, this happens for $d_{\text{votex}}≳\;70\;\text{nm}$ ($\approx 9.9\;l_{\text{ex}}$), consistent with previous reports.^[^ ^33^, ^34^, ^35^, ^36^, ^37^, ^38^, ^39^, ^40^ ^]^ This threshold exceeds the $d\geq 7.2\;l_{\text{ex}}$ predicted for ideal, isotropic spheres,^[^ ^29^ ^]^ underscoring the influence of crystalline anisotropy. While vortex formation lowers the demagnetizing energy, magnetic anisotropy imposes an energy penalty. The balance shifts in favor of the vortex only when the particle is sufficiently large for the demagnetizing energy to dominate, hence the larger critical size.

To isolate the anisotropy effects from those connected to exchange within this regime, we simulated a NF which preserves the realistic irregular morphology, but whose grains are fully exchange‐coupled (k=1). This case is shown in Figure 1C. As it can be seen, the vortex nucleates at larger sizes ($d_{\text{vortex}}>100\;\text{nm}$) compared to the k=0.25 case. This agrees with the scaling of the critical diameter with the exchange length, $d_{\text{vortex}}\propto l_{\text{ex}}=\sqrt{2A/\mu_{0}M_{s}^{2}}.$ The lowering of the intergrain exchange kA decreases the effective exchange coupling, shortens $l_{\text{ex}}$, and therefore decreases $d_{\text{vortex}}$ for the k=0.25 case. In other words, weakening the exchange coupling at the grain boundaries reduces the energetic cost of nonuniform spin textures, enabling the stabilization of the vortex state in smaller NFs.

The qualitative behavior of both k=0.25 and k=1 cases is, however, alike. In both cases, we observe a sharp rise of the coercivity just above the vortex nucleation threshold, as the magnetization becomes more vortex‐like with size. The sharper features of the vortex configuration are then pinned more easily by material disorder, which comprises pinning between grain boundaries (*k*) and/or the existence of a collection of random anisotropy axes within the particle, resulting in the increase of the coercivity. For both k=0.25 and k=1, the $H_{C}$ peaks at $H_{C}≅14\;\text{mT}$ within the vortex regime. Given this lack of dependence on the intraparticle pinning *k* for the occurrence of a maximum in the coercivity, we have cross‐tested the role of the random anisotropy axes within the particle against a reference system. As such, we have simulated a perfect nanosphere (NS) geometry with uniform uniaxial anisotropy $K_{u}∥z$. The results, included in Section S5, Supporting Information, reveal the formation of a vortex at $d_{\text{vortex}}>93$ nm, yet a constant drop of the coercivity until it reaches 0 mT for *d* = 107 nm. The next section examines how this trend affects the magnetization‐reversal mechanisms of NFs and NSs. For the present discussion, it suffices to note how the hyperthermia sweet spot depends sharply on structural disorder. Even when every grain is fully exchange‐coupled (k=1), the randomly oriented easy axes provide pinning potentials for the vortex, introducing internal energy barriers resulting in a nonzero coercivity and enhanced magnetic losses.

Beyond the “sweet spot diameter,” the coercivity of both NF cases decreases, following a power‐law decay, scaling as $d^{-1.5}$ for k=0.25 and $d^{-1.3}$ for k=1, in agreement with previously reported scalings for the vortex regime,^[^ ^30^, ^65^, ^66^, ^67^, ^68^, ^69^, ^70^ ^]^ and with the $H_{C}\propto K_{\text{eff}}\propto d^{-\frac{3}{2}}$ scaling consequence of the random‐anisotropy averaging.

In the next section, we show that magnetization reversal hinges on nanoparticle size. Two different mechanisms have been observed, which explain why the maximum of the coercivity is shifted from lower diameter *d* in the case of k=0.25 to larger *d* for the k=1 case.

### 2.2 Magnetization Reversal in the Vortex Regime

In Section 2.1 we investigated the coercivity as a function of NF size, finding a nonmonotonic trend within the vortex regime: an initial rise up to a critical $d_{\text{vortex}}$ size, followed by a decrease. By cross‐testing with the perfect sphere case NS (see Supporting Information S5), we found there a monotonous drop of the coercivity within the vortex regime until it reaches 0 for NS diameters above *d *= 107 nm. Interestingly, we observed in the NS that the vortex core points along the *z*‐axis at remanence up to *d *= 107 nm, delivering a nonzero coercivity and switching instantaneously. However, above *d *= 107 nm, the reversal goes through a gradual process in which the vortex core orients itself perpendicularly to the *z*‐axis, resulting in both zero remanence and coercivity. This change of the magnetization reversal mechanism obeys volumetric arguments: Starting from *d *= 108 nm, the fraction of magnetic moments forming the vortex core compared the whole moments in the nanoparticle is $V_{\text{core}}/V_{\text{NS}}<\frac{1}{3}$, i.e., the core is not the main volume. As such, the flux‐closure moments along the *x* and *y* directions carry more volume. The NS thus minimizes its energy by rotating the magnetization and aligning about half (a bit more due to deformation of the profile) of these flux‐closure spins with the field (*z*) direction, while the core turns perpendicular to *z*. As a consequence, the remanent magnetization and coercivity drop to zero.

**Figure 2:**
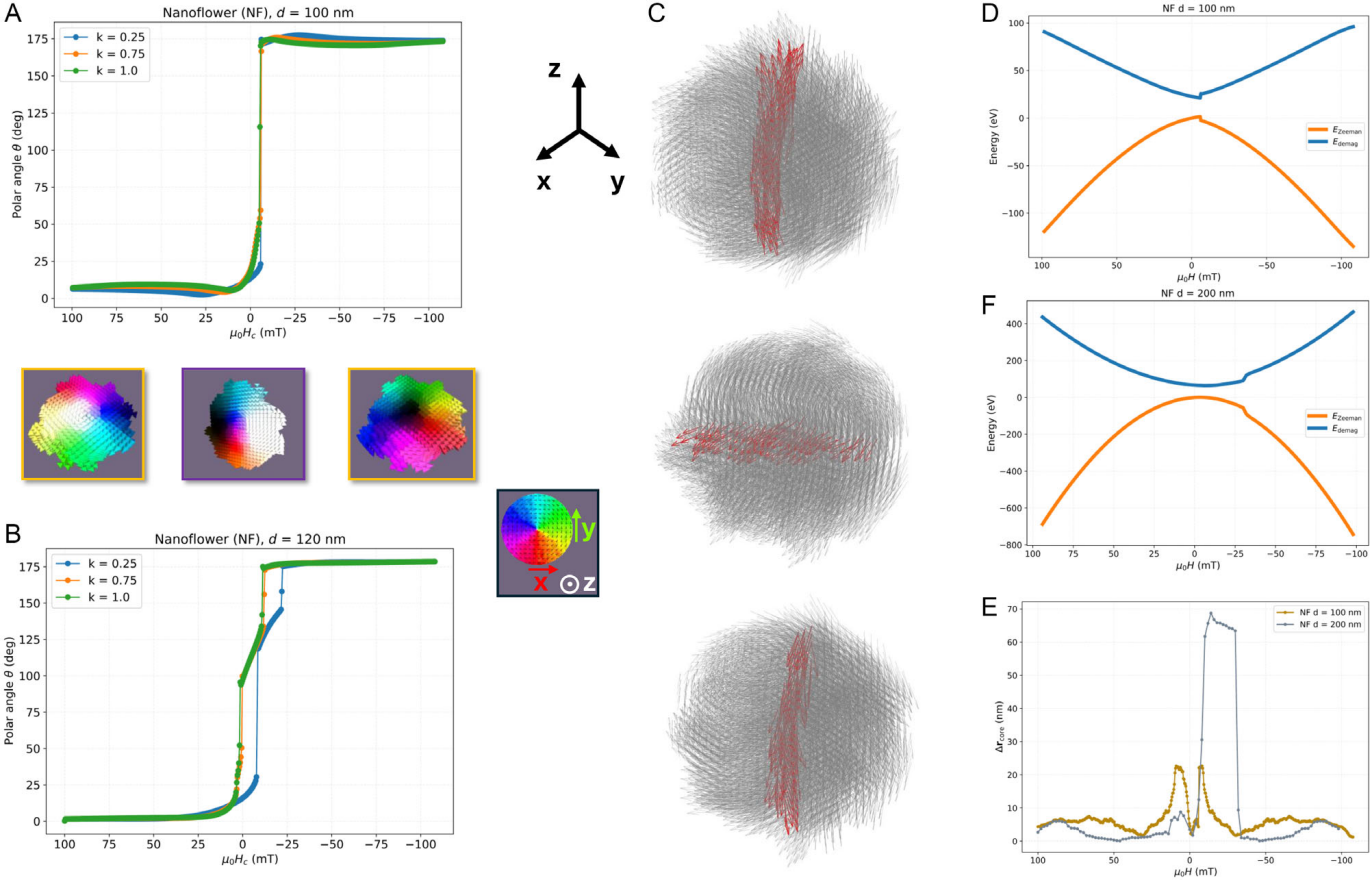
Elucidating the magnetization reversal mechanism of the vortex state. A,B) The polar angle *θ* of the vortex core direction versus applied magnetic field $\mu_{0}H_{z}$ for single realizations of (A) at *k* values of 0.25, 0.75 and 1.0, for (A) *d *= 100 nm and (B) *d *= 120 nm NFs. The snapshots represent the magnetization states: the left and right ones (with yellow border) show the vortex core along the positive (white core region) and negative (black core region) *z* direction, respectively, whereas the middle one with purple border has the core oriented in an in‐plane direction. The color wheel is included to help with the interpretation of these directions. C) The rotation of the vortex core for *d *= 110 nm. The core direction is marked in red and the flux‐closure cells in gray. D,E) The $E_{\text{demag}}$ and $E_{\text{Zeeman}}$ terms for *d *= 100 nm and *d *= 200 nm with k=0.25 as a function of field. Whereas for *d *= 100 nm, the $E_{\text{Zeeman}}$ is minimized by keeping the vortex core along *z*, for *d *= 200 nm, the $E_{\text{demag}}$ imposes the smooth reversal of the magnetization at the energy penalty of a core perpendicular to the field. This can be further verified in F) where the position of the core relative to the NF center, $\Delta r_{\text{core}}$, is plotted for both cases. A sudden displacement to the side of the vortex core, coinciding with the field range where the core is aligned perpendicular to *z*, enabling the flux‐closure moments to minimize the $E_{\text{Zeeman}}$, is detected.

In the NF, we find the same $V_{\text{core}}/V_{\text{NS}}=\frac{1}{3}$ threshold at which the switching mechanism shifts from one in which the core immediately reverses along the *z* direction to a rotation in which the core remains perpendicular to the *z*‐axis for a considerable field range. **Figure** **2**A,B depicts the polar angle *θ* between the net NF magnetization and the applied field direction +z for NFs with *d *= 100 nm (A) and *d *= 120 nm (B). We refer the reader to the technical details on how this angle is determined in S4, Supporting Information.

The situation illustrated in Figure 2A is representative of the range 70 < *d *< 110 nm, where the magnetization reversal process typically (Because we always investigate several realization of randomly generated geometries, both processes happen in an overlapping size range, and we identify the peak with the transition of which process dominates the resulting trend in the coercivity.) happens in one step from the positive +z to the negative −z direction (yellow‐marked snapshots) and spends only brief moments in intermediate states where the net moment lies perpendicular to the field (purple‐marked snapshots). This reversal mechanism corresponds to the windows where $\mu_{0}H_{C}$ is still rising, as shown in Figure 1.

In contrast, when the NF size exceeds *d *= 110 nm, the reversal process happens gradually. Figure 2B shows how the vortex core rotates into the plane and remains nearly perpendicular to the applied field (see snapshot in purple) over a wide field interval, from ≈−5 mT to ≈−25 mT before finally switching to −z. Both Figure 2A,B show that the two magnetization reversal processes are found regardless the *k* value. Within each process, there is also a weak *k* dependence, as the *θ* versus $\mu_{0}H_{z}$ curves for *k* = 0.25, 0.75, and 1 almost coincide.

In both regimes (core‐dominated and flux‐closure‐dominated), the anisotropy landscape of the NFs triggers a significantly different coercivity response compared to the NSs. Whereas the NS present a uniform $K_{u}$, in the NFs, as each grain has its own randomly oriented anisotropy easy axis, the magnetization can adjust locally, more or less aligning with the anisotropy of each grain almost irrespective of the vortex core's size and orientation within the total vortex volume. This means that in the NF, there is almost no reduction in the anisotropy energy barrier with size, as the magnetization will always find a local direction to align with. As *d* grows, and $V_{\text{core}}$ shrinks, but the number of grains (and thus, random directions) increases, the external field exerts a torque on the magnetization to overcome the switching energy barrier that is effectively decreasing in strength. As a consequence, the coercivity raises for the core‐dominated regime, whereas in the case of the NS, it drops continuously, consequence of the shrinking of the core volume, and thus, of the anisotropy barrier, with size.

The same applies to the flux‐closure‐dominated regime. Whereas in a NS with uniform $K_{u}$, the alignment of the vortex‐core perpendicular to *z* leads to zero net magnetization and hence vanishing coercivity, in NFs, however, the material grains pin the magnetization and delay the coercivity drop to larger *d* values, even though the reversal mode has changed. Within this second regime, we find $V_{\text{core}}/V_{\text{NS}}\propto d^{-1.4}$, consistent with the power law observed in Figure 1 for the decrease in coercivity beyond its maximum. The optimal balance between anisotropy and rotation is therefore reached at $V_{\text{core}}/V_{\text{NF}}=\frac{1}{3}$, where equal fractions of magnetization are aligned parallel and perpendicular (two directions) to the easy axes.

Figure 2C illustrates the mechanism for *d *= 120 nm with three representative snapshots, showing the vortex core (along the net magnetization direction, shown in red, surrounded by flux‐closure moments shown in gray) rotating from +z through an in‐plane orientation to −z. This sequence is identical for all NFs in this regime, irrespective of intraparticle pinning.

The different reversal mechanisms are further supported by the examination of the energy terms and the relative position of the vortex core with respect to the NF center of mass. Figure 2D,E includes the Zeeman ($E_{\text{Zeeman}}$) and demagnetizing energy ($E_{\text{demag}}$) terms corresponding to *d *= 100 nm (core‐dominated) (C) and *d *= 200 nm (flux‐closure‐dominated) (D). Indeed, for the smaller particles, the $E_{\text{demag}}$ is not so critical, and as such, the energy penalty of aligning the vortex core, which carries the net magnetization of the particle, perpendicular to the field, is high and undesirable. The NF magnetization rotates then strictly along *z*, at no intermediate position forming an angle with such a direction. However, for the larger NFs, once the $V_{\text{core}}/V_{\text{NF}}<1/3$, the energy penalty of aligning the core perpendicular to the field does not outweigh the gain of actually aligning the flux‐closure moments along the field, consequence of the larger $E_{\text{demag}}$. As such, the Zeeman energy term is minimized by aligning a fraction of flux‐closure moments along the field direction, while the $E_{\text{demag}}$ is sufficiently high to keep the vortex flux‐closure configuration. An energy penalty needs to be then paid for keeping the vortex core perpendicular to the applied field, but this is compensated by the satisfaction of $E_{\text{demag}}$ and $E_{\text{Zeeman}}$ constraints. Note how this is also reflected in the relative position of the vortex core with respect the NF center, as shown in Figure 2F. While the position of the vortex core keeps centered for the core‐dominated NFs, with a slight deformation at the magnetization reversal point, the larger ones are capable of moving the position of the core to reduce the $E_{\text{Zeeman}}$ term, increasing thus the volume parallel to the external field at the cost of the antiparallel volume. This mechanism is only relevant when the core lies perpendicular to the *z*‐axis.

Given the flower geometry, made of a collection of grains with random anisotropy axes, the anisotropy energy term, $E_{\text{anis}}$, averaged out from this random collection, has little influence to the reversal mechanism. Figure S5 in Section S6, Supporting Information, shows how this term acts like a background shifting the total energy, yet stays constant through the entire magnetization reversal.

To sum up, we conclude that the magnetization reversal mechanism is governed primarily by the size dependence of the vortex profile: when the vortex core dominates, switching proceeds via an immediate reversal; when the flux‐closure volume dominates, reversal occurs by reorienting the flux‐closure moments along the field and anisotropy axis (*z*), and the core perpendicular to it. The threshold for both mechanisms is $V_{\text{core}}/V_{\text{NF}}=\frac{1}{3}$, which determines which part of the nanoparticle encompasses more magnetic moments. We have also found that introducing intraparticle disorder (grains with random local easy‐axis orientations) increases the effective anisotropy, leading to superior coercivity. In the next Section 2.3 we will elucidate the influence of the exchange reduction at the grain boundaries *k* in the coercivity for both switching regimes.

### 2.3 Influence of Intergrain Exchange Stiffness

In Section 2.1, a shift of the $d_{\text{vortex}}$ threshold to smaller values for the disorder‐rich NFs (*k* = 0.25) highlighted a predominant role of intergrain exchange stiffness, modeled by the parameter *k*. In Section 2.2, we established that the magnetization reversal mechanism in both NF and NS vortex states is fundamentally dictated by geometric factors; essentially, the size dependence of the vortex core profile. We observed, however, that the difference in the anisotropy energy landscape between a perfect sphere with one easy axis (NS) and a NF consisting of a large number of randomly oriented grains (NF) changes the macroscopic behavior, namely, whereas the NS shows a monotonous drop of coercivity, a coercivity maximum is reached in the NFs. Indeed, the combination of both pinning and anisotropy at intraparticle level is essential to tune the coercivity‐size relationship, and thus, the “hyperthermia sweet spot.” In this section, we will connect the role of intergrain exchange stiffness, source of disorder, to the NF coercivity.

While *k* itself is a microscopic parameter governed by atomic‐scale interactions, and thus, practically not possible to engineer experimentally, we propose to control the crystallite size during the synthesis. This is a working and practical route of tuning the disorder, and consequently the windows of the hyperthermia peak.^[^ ^21^, ^43^, ^44^, ^71^ ^]^ By adjusting the growing conditions, it is possible to vary the size of the grains, which, at a fixed NF size,^[^ ^18^, ^49^, ^58^ ^]^ determines the amount of constituent grains *N*. These determine the density of grain boundaries, conversely, the magnetic pinning landscape, offering an experimentally feasible pathway to engineer the coercivity response.

**Figure 3:**
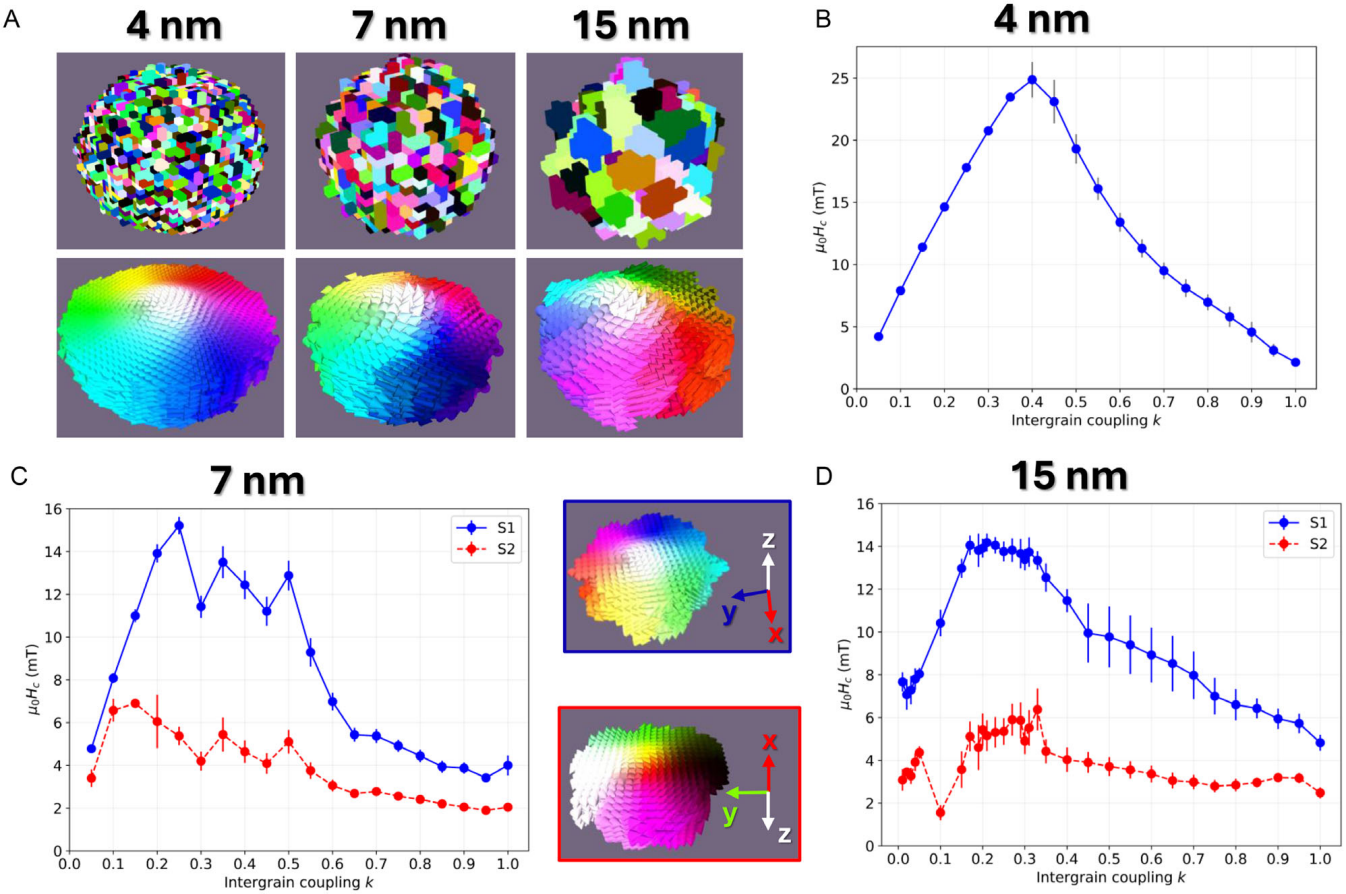
Influence of intergrain coupling *k* on the coercivity evaluated at three different grain sizes (4, 7, and 15 nm) in a *d *= 100 nm NF. A) Micromagnetic structure of the NFs: top shows the anisotropy distribution, each color representing a different anisotropy direction, and bottom, the magnetization of the NF at remanence. Note how the shape becomes more irregular as the grain size increases, which also implies the reduction of the total number of grains *N*. B–D) Coercivity versus intergrain coupling *k* for the 4, 7, and 15 nm grain‐sized NFs. Note in (C) and (D), the occurrence of two coercivity trends depending on the vortex core orientation at remanence: blue corresponds to the core oriented at remanence along the *z*‐axis (blue circles S1, top snapshot marked in blue), and red, perpendicular to the *z*‐axis (red circles S2, bottom snapshot marked in red). Data shown as mean ± SEM across seeds; using 20 seeds per *k*.

**Figure** **3**A shows snapshots of the studied grain sizes: 4, 7, and 15 nm for a fixed NF diameter of *d *= 100 nm, i.e., within the hyperthermia sweet spot. The selected grain size range used in our Voronoi tessellation is based on experimentally reported crystallite sizes in iron‐oxide NFs, e.g., refs. [19, 44, 45, 49], which reinforces the experimental relevance and general applicability of our results. The top row illustrates the anisotropy distribution, and it can be seen that the nanoparticle becomes more spherical and less irregular as the grain size is reduced. The bottom row illustrates a snapshot of the magnetization at remanence, corresponding to the vortex core oriented along the *z*‐axis. Figure 3B–D showcases the coercive field versus the intergrain coupling factor *k* for grain sizes of 4, 7, and 15 nm, respectively. The amount of grains decreases from *N* = 8181, 1527 to 155 from 4 to 15 nm, which implies the reduction of the grain boundaries, primary source of pinning. All three cases show a similar trend, although the cases of 7 and 15 nm show some particularities.

Starting with the common features, the coercivity describes an arch‐shaped pattern with a maximum achieved at intermediate exchange coupling strengths: k=0.40 for 4 nm grains, and a broadened regime of k=0.25−0.50 for 7 nm grains and k=0.15−0.40 for 15 nm. This trend reveals a subtle balance between the pinning and the coherence of the vortex profile. Starting from the case where the grains are strongly coupled (large *k*), the pinning potential wells become shallower: the strong exchange reduces both the pinning energy associated with individual defects and smooths the magnetization, averaging out over multiple pinning sites, further flattening the overall pinning landscape. As a result, the coercivity remains at a low value. Worth to mention, the abovementioned arch‐shaped pattern of the coercivity versus intergrain exchange coupling is also found in different grain‐textured systems, such as nanocomposites,^[^ ^72^ ^]^ and magnetite nanoclusters resembling NFs,^[^ ^73^ ^]^ showing it is a universal signature over different magnetic systems rich in pinning sites.

As the coupling decreases, the pinning potentials become deeper, meaning that each grain is almost decoupled from its surrounding, behaving like an individual entity. The magnetization does no longer average out over the grains, and as a consequence, the coercivity raises, describing a maximum that gets narrower as the amount of pinning sites increases significantly. When the grains are almost fully decoupled (typically below k=0.2), there is a very low exchange energy penalty for adjacent grains to have a misalignment in their magnetization, resulting in the loss of a rigid vortex profile. In this situation, such low coupling allows the (dipolar field assisted) switching of individual grains, resulting in a significantly lower coercivity. We have included results for the extreme case in S7, Supporting Information, of no intragrain boundaries (k=0). The coherence of the vortex profile is completely lost, and instead, a clear multidomain pattern, in which each grain behaves as an independent single‐domain particle with its own random easy axis. In this scenario, the random direction at each grain cancels out, and a zero coercivity, corresponding to a true multidomain structure, is found.

Figure 3C,D shows an additional red curve, with an almost constant value of the coercivity. Since *d *= 100 nm lies near the crossover between core‐dominated and flux‐closure‐dominated reversal, some realizations with larger grains (7 and 15 nm) switch through immediate core reversal (S1, as shown in the snapshot marked in blue), while others are flux‐closure‐domains dominated and display the vortex core pointing perpendicular to *z* at remanence (S2, shown also in the snapshot marked in red). As such, the realizations shown in S2 display a low and almost constant coercivity ($\mu_{0}H_{C}\approx 4\;\text{mT}$ and ≈6 mT, respectively), consequence of the rotational reversal mechanism, which leaves the vortex core pointing perpendicular to *z* at remanence, giving only a small net moment. This kind of reversal dynamics is less affected by grain boundaries, yielding the absence of any significant dependence on *k*.

Furthermore, the similar coercivity values for the 7 and 15 nm‐sized grains indicate a balance between the number of grains, *N*, and pinning density. In this way, for 7 nm grains, with a larger number of grains, the effective anisotropy is reduced as a consequence of averaging over more anisotropy directions, and so is the coercive field, as $\mu_{0}H_{C}\propto \;K_{\text{eff}}\propto \;N^{1/2}$. However, a larger number of grains *N* imply a larger total grain‐boundary area, which grows as $\propto N^{1/3}$, and thus, supplying more pinning sites. The similar (but not identical) weak scaling of these competing effects leaves the coercivity virtually unchanged across the two grain sizes.

By combining the impact of intergrain exchange stiffness on coercivity with the presence of two size‐dependent reversal mechanisms, the trends in Figure 1C (for *k *= 0.25 and *k *= 1) are readily understood. For loosely coupled grains (*k *= 0.25), which represent the case of maximized intraparticle disorder, the coercivity peaks near the boundary between the core‐dominated and flux‐closure‐dominated regimes. In this low intergrain‐coupling (strong‐pinning) configuration, coercivity is maximized when the number of constituent grains, and hence the dispersion of local anisotropy axes, is largest, provided that the reversal remains core‐dominated. In this configuration, the vortex core profile keeps coherent and experiences the maximum pinning, indeed, when the number of grains is maximized. Once the magnetization reversal becomes flux‐closure‐dominated (for *d* ≳ 107 nm), the reversal becomes almost independent of the intergrain stiffness. Instead, the coercivity is dictated by the interplay between the demagnetization energy, which grows with *d*, and the random‐averaging of anisotropy directions, which decreases with *d*. Consequently, the coercivity maximum is displaced toward larger NF sizes compared to the weakly coupled case and occurs near the turning point between these demagnetizing and anisotropy opposite trends. The underlying energy balances supporting these statements have been discussed in connection with Figure 2.

We can conclude from our results that the coercivity is largest at the intermediate *k* range, where the vortex remains coherent and encounters high energy barriers consequence of deeper pinning potentials. Larger grains (Figure 3D), with fewer boundaries, need stronger pinning to reach coercivities comparable to grains half the size (7 nm grains, Figure 3C), and thus, the *k* range for maximized coercivity is shifted to lower values. In the extreme case of 4 nm‐sized grains, the effective exchange (exchange over the entire nanoparticle) is weaker, rendering the vortex core profile sharper. The harder pinning boosts the coercivity up to almost twice the value (25 mT compared to 14 mT), compensating the loss of shape anisotropy consequence of their more regular (spherical‐like) morphology.

Our results highlight the key role of intergrain exchange (grain‐boundary pinning) in determining $\mu_{0}H_{C}$. This interplay helps to explain the high heating efficiency of NFs and other nanoparticles rich in intraparticle grain boundaries, compared to their defect‐free counterparts, as predicted by Lappas et al.^[^ ^22^ ^]^

## 3 Conclusion

In this work, we have provided the complete map of the coercivity versus size dependence for one of the most promising candidates for magnetic hyperthermia applications, magnetic NFs. Beyond the single domain SW regime, we describe the onset of a magnetic vortex, often referred to as multidomain phase. In NFs, this phase comprises a secondary maximum of the coercivity at sizes around 100–150 nm nanoparticle diameter, providing a window for maximized hyperthermia efficiency. We expect this maximum to be robust against dipolar interactions since the flux‐closure pattern does not generate stray fields.

We examined the reversal mechanism on both sides of the vortex coercivity peak and found two different processes. When the vortex core, which carries the NF's net magnetization, dominates the particle volume ($V_{\text{core}}/V_{\text{NF}}$ > 1/3), switching occurs via the immediate core reversal. For the larger NFs, once the flux‐closure region becomes the dominant fraction of the NF, reversal proceeds through a smooth rotational process, in which the core aligns perpendicularly to the applied field direction. As the NF diameter increases, the fraction of the volume taken up by the core shrinks, the net magnetic moment falls, and the coercive field decreases, following a power law $d^{-\alpha }$ with α≈1.5–1.3; the decay steepens as intraparticle disorder decreases. The experimental observation of such a distinct reversal mechanism will be made possible by noninvasive local probe techniques, such as Nitrogen‐Vacancy SPM or Magnetic X‐ray microscopy, which have recently been demonstrated to resolve the individual signal of MNPs.^[^ ^74^ ^]^

We identified the interplay between two features of the grains typical for the NF morphology as the source of the secondary peak in the coercivity. The combination of randomly oriented grain easy axes and weakened intergrain exchange creates pinning potentials that hinder core reversal, producing a coercivity peak that is absent in nondisordered spherical nanoparticles. Our simulations do not account for surface anisotropy. Note that this is typically relevant for high surface‐to‐volume ratio configurations (few nm, e.g., ref. [75]), which are almost two order of magnitude smaller than the particle sizes where the vortex texture is accommodated (≳100 nm). We also modeled how changing grain size, an experimentally accessible way to tune intergrain boundaries, affects coercivity. Halving the grain size doubles the boundary area, boosting the pinning, yet this effect is counteracted by the larger number of grains, thus averaging their random easy‐axis orientations and lowering the effective anisotropy. Even though the size of the coercivity peak does not depend on the material disorder, its position does, and is shifted from 150 nm for fully coupled grains to 110 nm for weakly coupled ones. Despite the fact that the exchange coupling cannot be experimentally tuned, engineering the grain size in the NF synthesis is possible and provides a certain degree of tunability to tailor the hyperthermia window, as a larger number of grain boundaries with relatively weak coupling reduction have the same effect on the effective exchange as a lower number with stronger coupling reduction.

Our findings clarify why NFs excel as hyperthermia agents. Their unique morphology, rich in pinning sites and local random anisotropy directions, results in favorable stabilization of a vortex beyond the SW limit, triggering a secondary peak in their coercivity, absent in the typical nanoparticles, but that allows the NFs to perform efficiently within a size range above the single‐domain limit. We have also shown that the coercivity has a direct equivalent in the SAR, validating our trends with the available experimental data. Our findings advance the understanding of spin disorder and position vortices as excellent nanoheaters, as they are large enough to offer high thermal stability while being less prone to agglomeration, both critical factors influencing the heating performance of nanoparticles.^[^ ^11^, ^25^, ^26^ ^]^ Furthermore, the specifics of the material disorder, like grain size, allows to finetune the size range of this regime, thereby optimal heating can be achieved in a broad range of sizes. We anticipate that the insights into the fundamentals of the nanoscale magnetization will guide the design of even more effective NFs for magnetic hyperthermia applications.

## 4 Methods Section

Micromagnetic simulations were performed using Mumax3.^[^ ^76^ ^]^ The NFs were generated from a perfectly spherical geometry with an initial diameter of *d *= 10–400 nm. A Voronoi tessellation was applied to this sphere to define discrete regions,^[^ ^77^ ^]^ corresponding to individual material grains. The grains with Voronoi centers laying inside the base sphere were kept in their entirety (also extending outside the sphere), whereas areas of grains whose Voronoi center lays outside of the sphere were completely discarded. This results in the nonspherical flower shape as shown in Figure 1. We checked that the average volume of the randomly generated geometries coincides with that of the sphere, thus resulting in the same effective diameter. Each grain was assigned a randomly oriented anisotropy axis to reproduce the polycrystalline nature typically observed in experimentally synthesized NFs.^[^ ^19^, ^44^, ^45^, ^48^ ^]^ To reproduce the effect of intraparticle disorder, the magnetic coupling between adjacent grains was modeled by rescaling the exchange parameter *A* at the grain boundaries by a factor *k* lying between 0 and 1. We find good agreement with the experimental results for k=0.25.^[^ ^19^, ^44^, ^48^, ^49^ ^]^ Material parameters typical for iron oxide were used based on the literature. These include saturation magnetization $M_{s}=400\times 10^{3}\;A\;m^{-1}$,^[^ ^78^, ^79^ ^]^ exchange stiffness $A=10\;\text{pJ}\;m^{-1}$,^[^ ^80^ ^]^ and uniaxial magnetocrystalline anisotropy $K_{u}=10^{4}\;J\;m^{-3}$.^[^ ^59^, ^78^, ^81^, ^82^ ^]^ Each simulated size has been realized 20 times with a different random seed for the Voronoi tessellation to ensure the statistical significance of our results. Extended information is included in S1, Supporting Information.

### 4.1 Statistical Analysis

#### 4.1.1 Preprocessing

No preprocessing was applied. Raw outputs from each simulation run were used as produced by the Mumax3; no transformations or outlier removal were performed. Each random seed contributes one independent observation to the statistics.

#### 4.1.2 Data Presentation

Unless stated otherwise, results are reported as mean ± SEM (standard error of the mean) computed across independent runs with different random seeds for a given condition.

#### 4.1.3 Sample Size (n)

For all aggregate quantities, *n* denotes the number of independent runs (distinct random seeds) per condition.

**Figure 1:** n=20 runs for each nanoparticle diameter from 10 to 400 nm in 10 nm increments (i.e., 40 sizes, 2 *k* values; total of 1600 runs).

**Figure 3:** For each grain size ($d_{\text{grain}}=4$, 7, and 15 nm) and for each intraparticle pinning value *k* from 0.05 to 1.00 in steps of 0.05, we performed n=20 runs (distinct seeds) per $\left(d_{\text{grain}},k\right)$ pair.

#### 4.1.4 Statistical Methods

Because this is a simulation study and variability arises solely from stochastic initialization (random seeds), uncertainty is summarized by the SEM across seeds.

#### 4.1.5 Software

Analyses were performed in Python (NumPy, pandas, SciPy/StatsModels, Matplotlib). Simulations were performed using Mumax3, as indicated in the previous section, via scripts that randomize seeds reproducibly. Postprocessing scripts aggregate per‐seed outputs and compute means and SEM.

## Supporting Information

Supporting Information is available from the Wiley Online Library or from the author.

## Conflict of Interest

The authors declare no conflict of interest.

## Supporting information

Supplementary Material

## Data Availability

The data that support the findings of this study are available in the Supporting Information of this article.
