# Supplementary material for: Coercivity‐Size Map of Magnetic Nanoflowers: Spin Disorder Tunes the Vortex Reversal Mechanism and Tailors the Hyperthermia Sweet Spot
Source: Small Sci. 2025 Nov 5;6(1):e202500490. doi: 10.1002/smsc.202500490 (PMC12825459; doi:10.1002/smsc.202500490)
Supplement: Supplementary file 1 — Supplementary Material [file SMSC-6-e202500490-s001.pdf]

Supporting Information to: Coercivity-size map of magnetic nanoflowers: spin disorder tunes the vortex reversal mechanism and tailors the hyperthermia sweet spot

Elizabeth M Jefremovas Lisa Calus Jonathan Leliaert**

Dr. E. M. Jefremovas
Department of Physics and Materials Science, University of Luxembourg, 162A Avenue de la Faiencerie, L-1511 Luxembourg, Grand Duchy of Luxembourg
Institute for Advanced Studies, University of Luxembourg, Campus Belval, L-4365 Esch-sur-Alzette, Luxembourg
Email Address: elizabeth.jefremovas@uni.lu
L. Calus
DyNaMat, Department of Solid State Sciences, Ghent University, 9000 Ghent, Belgium
Prof. Dr. J. Leliaert
DyNaMat, Department of Solid State Sciences, Ghent University, 9000 Ghent, Belgium
Email Address: jonathan.leliaert@ugent.be

Keywords: *Magnetic hyperthermia, Iron oxide nanoparticles, Spin disorder, Nanomagnetism, Micromagnetic simulations*

S1 Simulation details

Numerical simulations were performed using Mumax3 [1], an open-source GPU-accelerated micromagnetic simulation program. The software solves the time- and space- dependent magnetization evolution, described by the Landau-Lifshitz-Gilbert equation, in nano- to micro- scale magnets using a finite-difference discretization. We refer to Ref. [1] for a detailed description of the physical model implemented, and instead focus below on the technical details of the simulations we performed.
The nanoflowers were generated from a perfectly spherical geometry with an initial diameter of $d = 10 - 400$ nm. A Voronoi tessellation was applied to this sphere to define discrete regions [2], corresponding to individual material grains. The grains with Voronoi centers laying inside the base sphere were kept in their entirety (also extending outside the sphere), whereas areas of grains whose Voronoi center lays outside of the sphere were completely discarded. This results in the non-spherical flower shape as seen in the inset of Figure 1 of the main paper. We checked that the average volume of the randomly generated geometries coincides with that of the sphere, thus resulting in the same effective diameter. Each grain was assigned a randomly oriented anisotropy axis to reproduce the polycrystalline nature typically observed in experimentally synthesized nanoflowers [3, 4, 5, 6].
To reproduce the effect of intra-particle disorder, the magnetic coupling between adjacent grains was modeled by rescaling the exchange stiffness parameter A at the grain boundaries by a factor k lying between 0 and 1. We find good agreement with the experimental results for $k = 0.25$ [4, 3, 7, 6]. Material parameters typical for iron oxide were used based on literature. These include saturation magnetization $M_s = 400 \times 10^3$ A/m [8, 9], exchange stiffness $A = 10$ pJ/m [10], and uniaxial magnetocrystalline anisotropy $K_u = 10^4$ J/m^3 [11, 12, 13, 8]. Each simulated size has been realized between 15-25 times with a different random seed for the Voronoi tesselation to ensure the statistical significance of our results.
We have adjusted the cell size and number of cells depending on the nanoflower size, the grain texture and the material parameters. First, the exchange length of our material is $l_{ex} = \sqrt{\frac{A}{\mu_0 M_s^2}} \cong 7.052$ nm, setting the upper limit for our cell size. We have benchmarked a collection of cell sizes from 1.500 to 5.000 nm in steps of 0.125 nm, finding, for the same field step, a factor 3 performance difference between the smallest and the largest discretization cells. Considering the amount of simulations performed in this

work, we fixed the cell size as a function of the nanoflower diameter to maximize the compromise between accuracy and computing time. The number of cells was varied depending on the nanoflower diameter to keep the grid size dimensions *7-smooth*. The values and cell dimensions are included in the following table.

Table S1: Nanoflower diameter, cell size and number of cells N used for each realization

d (nm)	cell size (nn)	N
10 – 15	1.125	16^3
17 – 20	1.125	24^3
22 – 30	1.125	32^3
32 – 48	1.125	48^3
50 – 70	3.125	32^3
72 – 100	3.125	48^3
110 – 140	5.000	32^3
150 – 200	5.000	48^3
210 – 280	5.000	64^3
290 – 400	5.000	128^3

For $d \leq 50$ nm, the grain size was reduced from 15 nm to 5 nm, setting one random anisotropy direction for all grains (all grains following the same K_u) with fixed $k = 1$, to reproduce the single-domain behavior.
In addition to the nanoflower geometry, we have also simulated the case of idealized spherical particles with uniform geometry, no grain boundaries, and a single anisotropy axis (K_u) aligned along z. The realizations follow the same cell size and N indicated in Table S1.
The main parameter shown in this study is the coercive field $\mu_0 H_C$, which is extracted from the hysteresis loop. For simulating these loops, we initialized the magnetization at saturation ($\mu_0 H_z = 1.2$ T). The field is then reduced in a stepwise fashion to $\mu_0 H_z = -0.1$ T, first in steps of -2.5 mT from saturation to $\mu_0 H_z = 0.1$ T, and then, in finer steps of -0.1 mT to fully capture the switching of the magnetization. In each step, we minimize the total energy of the system by using the *relax()* function of Mumax3, which solves the Landau-Lifshitz-Gilbert equation without precession.

S1.1 Simulation hardware

Most simulations were run on the Tier 1 supercomputer (Hortense) of the Flemish supercomputer center within a Tier-1 compute project. The simulations consumed 40,000 GPU-hours on 80 NVIDIA A100 GPU's over an 8 month period. In addition, we made use of the Tier 2 GPU infrastructure of both Ghent University and the University of Luxembourg, both providing NVIDIA V100 GPU's, and the Tier 1 Luxembourg national supercomputer MeluXina, providing NVIDIA A100 GPU's.

S2 Hysteresis loops (selected sizes)

Figure S1 includes the complete hysteresis loops for $d = 30$ nm, $d = 70$ nm, $d = 100$ nm, and $d = 120$ nm. 20 different random initial configurations per NF size are performed to ensure that the average result is statistically significant. The middle panel includes the average of the seeds, from which the coercivity is extracted. On the right hand side panel we have included the equatorial planes for each configuration at remanence. The evolution from single domain ($d = 30$ nm) to domain wall ($d = 70$ nm) and a vortex pointing along the z direction ($d = 100$, and 120 nm) can be inspected.
To quantify the magnetic energy loss associated with the reversal process, we evaluated the area enclosed by the hysteresis loops. The enclosed area was obtained by numerical integration of the magnetization curves using the trapezoidal rule, applied separately to the ascending and descending branches of the loop. The difference between these integrals yields a positive measure of the loop area, which directly reflects the dissipated energy density, $\mathcal{E}$. This magnitude can be compared to the Specific Absorption Rate

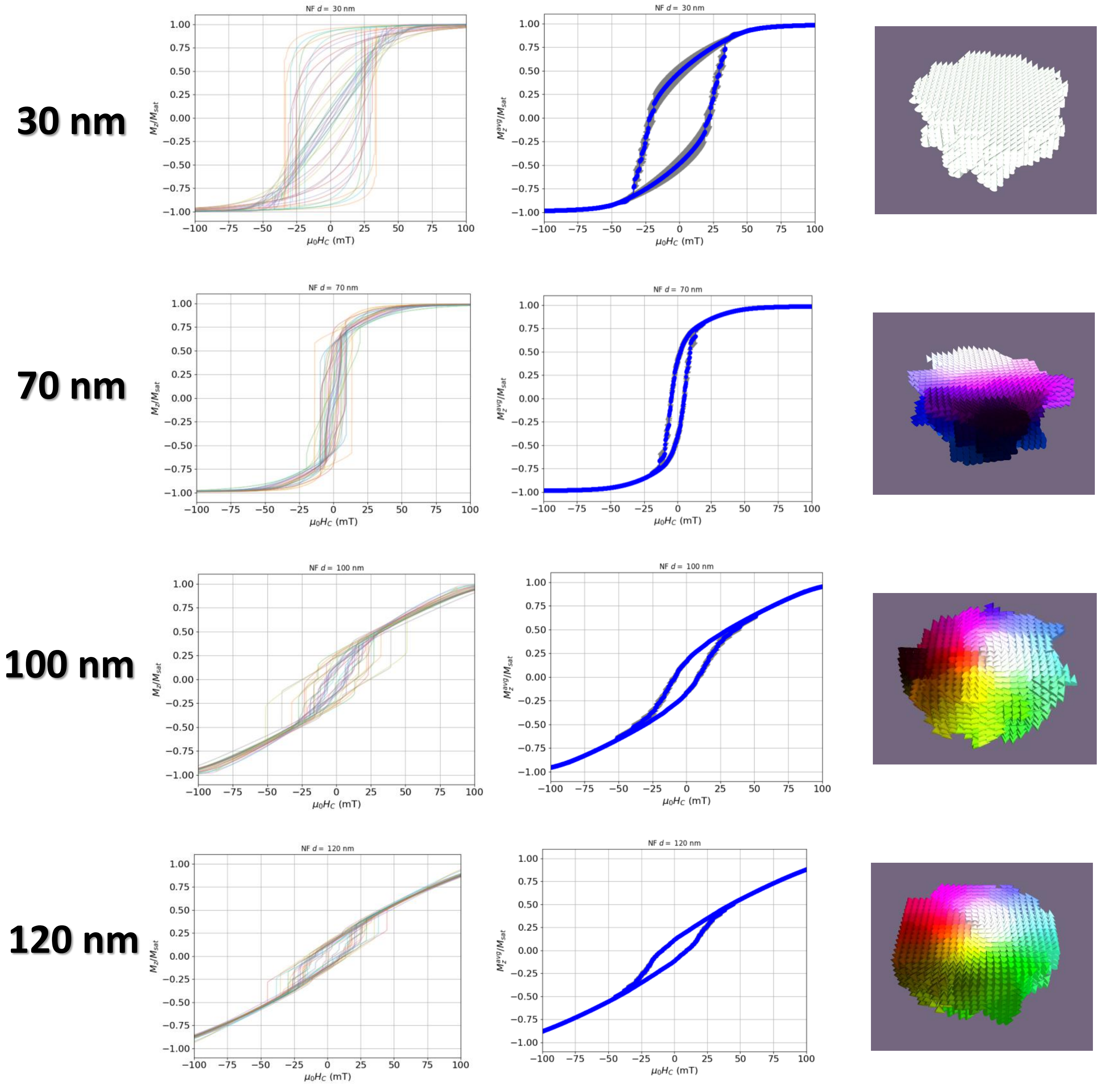

Figure S1: Hysteresis loops for d = 30, 70, 100, and 120 nm NFs. Left column includes the loops of the 20 different realizations, each of them with a different random seed. The central column represents the averaged hysteresis loop from the individual realizations. The coercivity for each size in Fig. 1 of the main text is extracted from the averaged loops. Left column includes equatorial cuts of the remanent magnetization, illustrative of single domain (d = 30 nm), domain wall ($d = 70$ nm) and vortex ($d = 100$ and 120 nm).

NF d (nm)	$\mathcal{E}$ (kJ/m^3)	e (mJ/g)	SAR (W/g)
30	20±3	4.0±0.6	1200 ± 180
70	4.7±0.8	0.82±0.17	290± 60
100	5.4±1.3	1.1±0.3	330 ± 90
120	4.0±0.8	0.82±0.20	250 ± 50

Table S2: Energy losses per cycle for a selected NF sizes, expressed as volumetric energy density ($\mathcal{E}$, in $\mathrm{kJ\,m^{-3}}$) and as specific energy per unit mass (e, in $\mathrm{J\,g^{-1}}$). The values of e were obtained by normalizing $\mathcal{E}$ with the mass density of maghemite ($\rho = 4.85\ \mathrm{g\,cm^{-3}}$, [14]). The values for the SAR are estimated at $f = 300$ kHz by multiplying $e \times f$.

(SAR), that measures the heating efficiency of the nanoparticles excited under an alternating magnetic field, and is described as:

$$SAR = \frac{f}{c} \times \oint \mu_0 M dH \quad (1)$$

being f the frequency of the AC-field excitation and c the concentration of magnetic nanoparticles. Hereby, both magnitudes can be compared, provided $\mathcal{E}$ is multiplied by the maghemite density $\rho = 4.9$ g/cm^3 [14]. In such a case, the energy losses become comparable to the value of the SAR normalized by the frequency f.

The values indicated in Table S2 are in agreement with the ones reported in the literature for maghemite multi-core nanoparticles. More precisely, the SAR value estimated for the single-domain NFs ($d = 30$ nm) is in good agreement with the experimental result of $SAR = 930$ W/g reported for 45 nm NFs under $f \cong 300$ kHz, and in range with the one reported for single-domain nanoflowers between 10-50 nm under $f \cong 100 - 500$ kHz [15]. Above the single-domain regime, the decrease of energy losses mimics qualitatively the case of maghemite ferrimagnetic nanocrystal assemblies of $d \approx 80 - 100$ nm, where a value of $SAR \cong 180 - 250$ W/g is reported [16]. Note that, for the experimental case, the heat release suffers from the nanoparticle agglomeration [17, 18], which yields a reduction in the energy losses compared to our simulated values for a single nanoflower.

A word of warning should however be raised when comparing the results obtained from the simulations and the experimentally reported ones. Given the nature of both worlds (quasistatic DC, dynamic AC of hyperthermia experiments), a direct 1:1 mapping of the coercivity into the SAR cannot be made, although a very reasonable estimate can be achieved, as well as the same qualitative behaviour is guaranteed.

Finally, it is important to recall that, within the vortex regime and at the typical frequencies employed in hyperthermia (kHz range), the dominant mechanism of magnetization dynamics is neither Néel nor Brownian relaxation. Instead, the response evolves from thermal diffusion to field-induced crossing of energy barriers, giving rise to a hysteretic behavior [19, 20, 21, 22]. Thereby, the main source of heat dissipation are hysteresis losses, with the coercivity serving as a reliable proxy to estimate thus the nanoparticle heating performance.

These arguments validate the use of the coercivity as a proxy for the heat generation of the nanoparticles.

S3 Single grain model validation for small nanoflowers

In Figure 1 of the main text, we showed the results of the simulations modeling the single-domain region with a single grain nanoflower, meaning, $N = 1$, which comprises the whole nanoflower. This procedure is consistent with the single-domain nature of the NF. If instead, the granular texture is maintained, with a fixed grain size of 15 nm, the obtained results are compatible with the ones of single domain, but indeed, with an increased noise. Figure S2 **(A)** includes the results, considering a fixed grain size of 15 nm, for the coercivity as a function of the NF diameter d. As it can be observed, within the error bars, the results corresponding to the single domain region ($d < 50$ nm) are compatible with the ones included

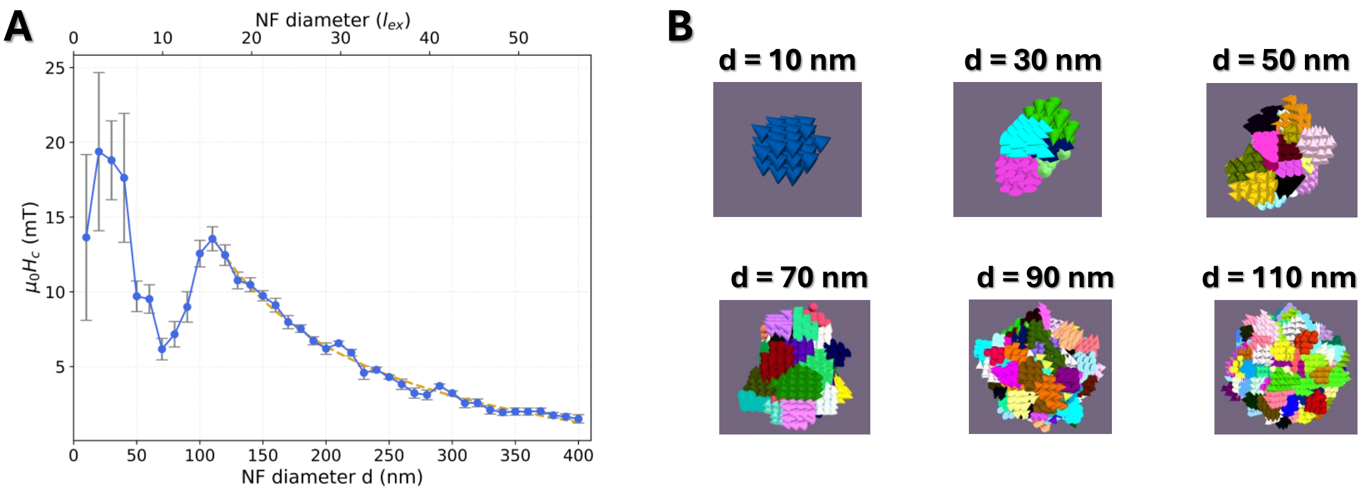

Figure S2: **(A)** Coercivity as a function of NF diameter for all realizations with 15 nm grains. For diameters below 50 nm the error bars exceed 40 % because the nanoparticle is too small to accommodate 15 nm grains and maintain a quasi-spherical shape, occasionally yielding empty or incomplete geometries. **(B)** Representative NF geometries at the indicated diameters; colored arrows show the uniaxial anisotropy direction of each grain. The geometries up to $d = 70$ nm appear unphysical, whereas the bottom row samples are sufficiently large to be unaffected by this artifact.

in the main text when a single-grain is considered, yet they carry a non-negligible noise due to the different geometry realizations. Indeed, the irregular shape of the NFs, together with the grain size itself (15 nm) make it impossible to accommodate all grains without sharp cuts, and also result in relatively large volume differences between different realizations of the geometry. As a consequence, some of the realizations do not display a nanoflower morphology, but rather unphysical "incomplete grain assemblies", as can be seen in the snapshots included in Figure S2 **(B)** for sizes up to $d = 70$ nm. As a reference, the ones for $d = 90$ and110 nm conform much more to the real nanoflower geometries.
To avoid any effects of unphysical geometries, we modified our simulations to model the single-domain region without an internal grain structure.

S4 Ratio vortex core *vs.* nanoparticle volume

Figure 2 **(E)** of the main text shows the ratio between the vortex core volume *vs.* total nanoparticle volume. We found a change in the magnetization reversal modes when the core volume fell below 1/3rd of the total volume.
To obtain the total volume of the nanoflowers with irregular geometry, we accessed the magnetization vector $\vec{m} = (m_x, m_y, m_z)$ at each simulation cell and multiplied the non-zero vectors by the corresponding cell size.
To quantify the core volume, we proceed in three steps. First, we average all cell magnetization vectors and normalize this average to define the unit vector $\hat{m}_{\text{net}}$, representing the overall magnetization direction. Next, for every cell we take the dot product between its magnetization $\vec{m}$ and $\hat{m}_{\text{net}}$; this yields a weight from -1 to +1 that measures how closely the cell aligns with the net direction. Cells with negative weights are counter-aligned, so we set their contribution to zero. Finally, we sum the volumes of the remaining cells, each multiplied by its positive weight, thereby obtaining an estimate of the vortex-core volume.

S5 Magnetization reversal in perfect spheres

In Figure 2 of the main text, we elucidate two regimes for the magnetization reversal, depending on whether the core or the in-plane flux-closure configuration dominates over the magnetic structure. This dual mechanism becomes particularly evident in the idealized case of a perfect sphere (NS) with a single uniaxial

anisotropy direction K_u along the field direction z, where the absence of intra-particle randomness allows to obtain exact results.

Figure S3 includes the coercivity **(A)**, remanent magnetization component along the field direction, m_z (**C**) and the relative vortex core volume V_{core}/V_{NS} (**D**), as a function of the NS diameter. Representative remanent states are shown in **(B)**.

Figure S3**(A)** plots the coercivity versus the NS diameter. Three distinct regimes can be detected. For $d < 93$ nm the nanosphere magnetization remains in a single-domain state, with uniform magnetization along $+z$, and $\mu_0 H_C = 60$ mT. This result is slightly above the prediction of the Stoner Wohlfarth model ($\mu_0 H_C = 2K/M_s = 50$ mT). We verified that this mismatch can be ascribed to the grid anisotropy [23], which is expected for such highly symmetric perfect NS. The single domain to vortex transition occurs at a larger diameter than the theoretical estimate $d_{vortex} \approx \sqrt{2A/\mu_0 M_s^2} \approx 70$ nm $\approx 10 \times l_{ex}$[24, 25, 26, 27, 28, 29, 30, 31]. The shift arises from the strong uniaxial anisotropy K_u, which disfavors the in-plane flux-closure pattern of the vortex and therefore requires a larger diameter before the demagnetizing energy can overcome the anisotropy energy.

Beyond the single domain range, $d \geq d_{vortex} = 93$ nm the NS adopts a vortex state, revealing the threshold size for which the demagnetizing energy dominates exchange and anisotropy. Once the vortex is stabilized, we distinguish two different regimes, with a transition at $d = 107$ nm. Within the first regime ($93 < d < 108$ nm), the vortex core points along z at remanence (see **(B)** and yellow inset), where the core points along z and is encircled by in-plane flux-closure moments. As d grows, the core fraction V_{core}/V_{NS} shrinks and $\mu_0 H_C$ falls accordingly. Both the remanent m_z and V_{core}/V_{NS} decrease $\propto d^{-4.63}$ (red line in **C**), which is steeper than the $\propto d^3$ rise of V_{NS}. This indicates that the core volume V_{core} itself is *shrinking* with increasing size, as $d^{-4.63}$ is steeper than the d^{-3} volumetric factor. Note that, for the NS, this shrinking core translates into a lower coercivity, whereas it *increases* the coercivity for NFs (see Fig. 1 of the main text). This opposite size dependence can be understood by considering the different anisotropy landscapes in NS and NF. In the perfect NS, the anisotropy is uniform with its easy axis parallel to the z axis. The vortex core is stabilized along this direction by an energy proportional to the anisotropy constant K and its volume V_{core}. As d increases, the core volume shrinks, and consequently, the anisotropy barrier falls. The Zeeman energy decreases too, because only the shrinking fraction of spins along the z axis can switch from an energetically unfavourable to a favourable orientation. As a consequence, the anisotropy barrier drops even faster than the volumetric d^{-3}, since the smaller the core, the larger the fraction of spins oriented in-plane, which do not contribute to the anisotropy. This large reduction in anisotropy energy barrier therefore explains the decrease in coercivity for NS.

The second regime starts as soon as the magnetization reversal is no longer core-dominated, which happens when $V_{core}/V_{NS} < \frac{1}{3}$. As observed in Figure S3**(D)**, this happens for diameters $d > 107$ nm, when the vortex core is no longer the main volume and the flux-closure moments along the x and y directions carry more volume. The NS thus minimizes its energy by rotating the magnetization and aligning about half (a bit more due to deformation of the profile) of these flux-closure spins with the field (z) direction, while the core turns perpendicular to z (see Figure S3**(B)**). As a consequence, the remanent magnetization and coercivity drop to zero. The balance between the energy terms, shown in the next figure S4, further supports this claim.

The deformation of the vortex profile also explains the gaps between the blue, yellow, and purple regions in Figures S3**(C)** and **(D)**. While the core is aligned with the easy axis, it enlarges slightly as the gain in anisotropy energy outweighs the cost in demagnetizing energy, so this regime extends to larger d than it would in the absence of uniform anisotropy $\parallel z$. After the core rotates perpendicular to the axis, its size is slightly suppressed, and also the volume in the different flux-closure directions is no longer equivalent: the volume parallel to the easy axis grows at the expense of the energetically less favorable one.

To further address this size-dependent magnetization reversal mechanism, we will study the case of NS of three different sizes, $d = 100$ nm (core-dominated reversal), and $d = 110$ nm and $d = 150$ nm, right close to the transition size entering the flux-dominated reversal mechanism, and well above the transition size. Figure S4**(A)** tracks the ratio V_{core}/V_{NS} as a function of the applied field ($+z$ direction) for the three cases, plotted in dark-yellow circles, purple squares, and grey diamonds, respectively. In all

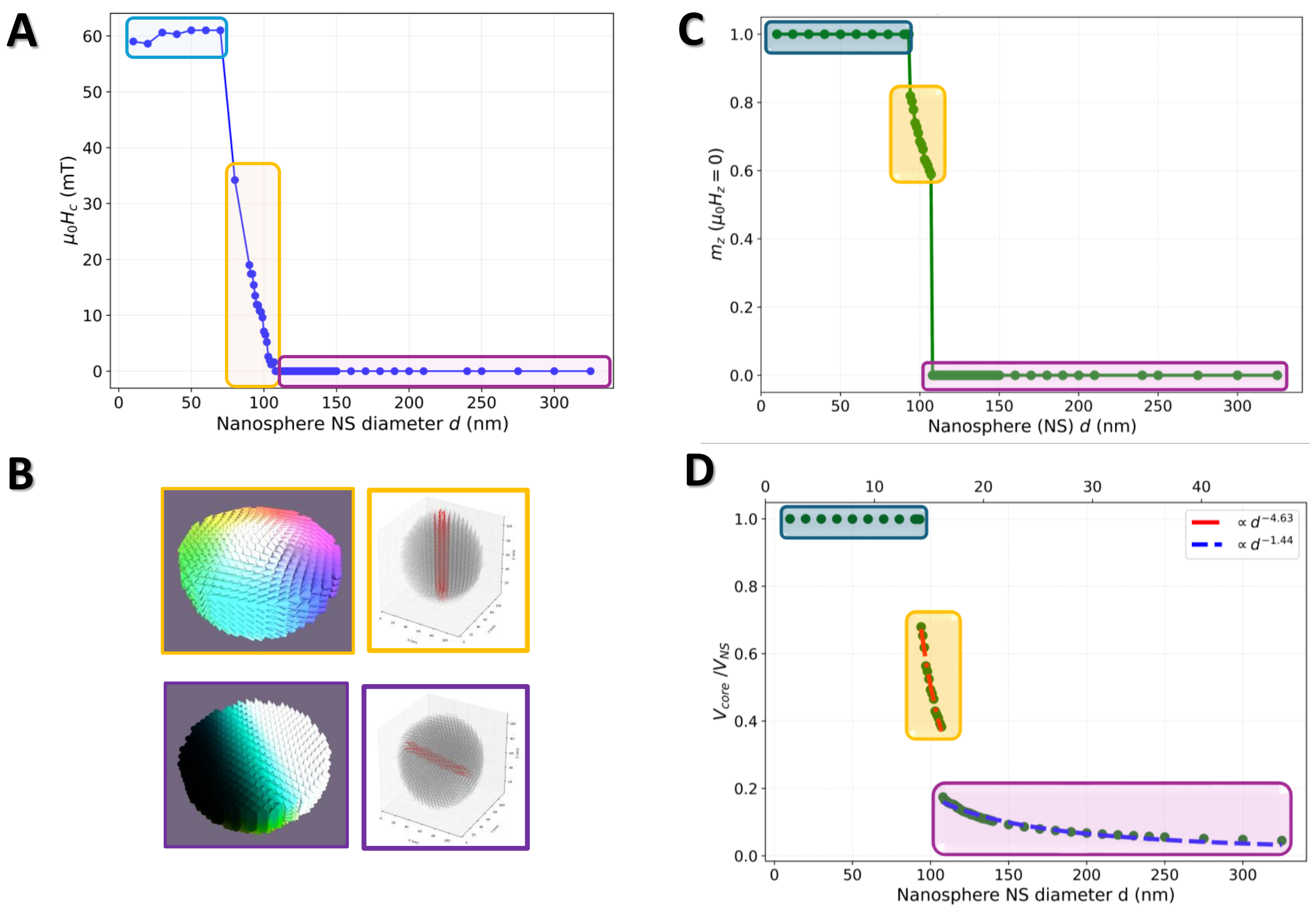

Figure S3: **(A)** Coercive field H_C; **C** magnetization along z at remanence; and **D**, V_{core}/V_{NS} ratio as a function of the NS diameter, revealing three regimes: single domain (blue), vortex with core along z (dark-yellow), and vortex with core in-plane (purple). The blue-shaded area denote the single-domain state. In the yellow region, the vortex core aligns with the z-axis at remanence, whereas in the purple region, the core lies within the XY plane. **(B)** snapshots at remanence from the equatorial plane of the magnetization for $d = 100$ nm and $d = 110$ nm, respectively.

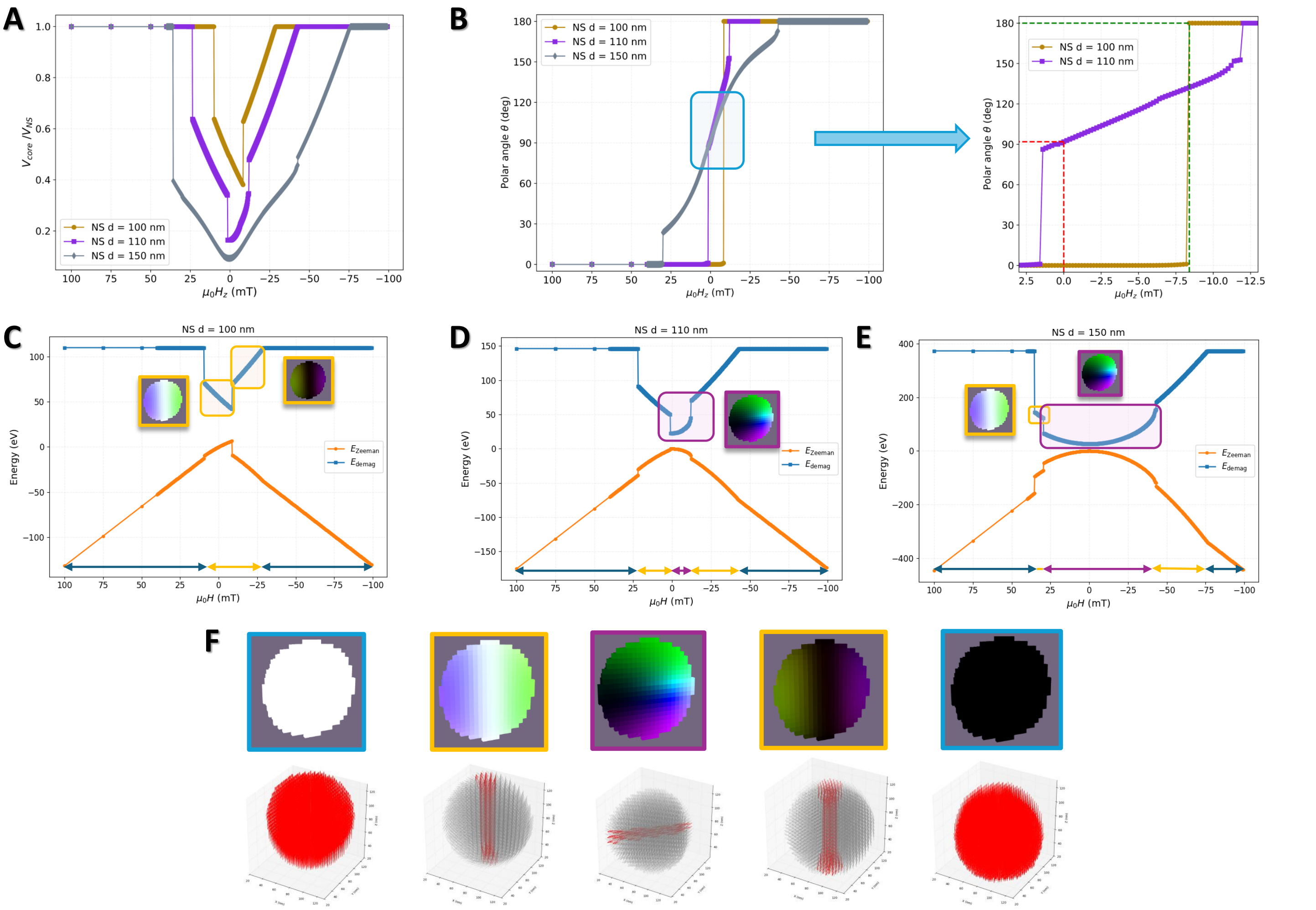

Figure S4: **(A)** Field dependence of the volume ratio V_{core}/V_{NS} for nanospheres with $d = 100$ nm (dark-yellow dots), $d = 110$ nm (purple squares) and $d = 150$ nm (gray diamonds); a pronounced drop near remanence appears for $d \geq 108$ nm. **(B)** Polar angle θ between the vortex core and the field axis as a function of the applied field $\mu_0 H_z$ for the same cases as in **(A)**. The smaller sphere reverses by a sharp flip of the core along z. The right-hand side inset zooms-in the region close to remanence, marking by a green dashed line the switching of $d = 100$ nm at $\mu_0 H_z \cong -8.4$ mT; and a red dashed line marking the $\theta \cong 90^\circ$ orientation of the net magnetization for $d = 110$ nm at remanence which results in the zero coercivity of above $d \geq 108$ nm. **(C)-(E)** E_{demag} and E_{Zeeman} energy terms plotted for $d = 100; 110$ and 150 nm-sized nanospheres, respectively. The yellow-shadowed frames indicate the regions where the vortex core is along the z direction, as indicated in the snapshots. The purple-shadowed regions, where the core is perpendicularly aligned to z, as well as the corresponding snapshots. **(G)** Magnetization snapshots cuts along the $y = 0$ plane for $d = 110$ nm. Left and right extremes picture the saturation along $+z$ (white) and $-z$ (black), respectively. The left snapshot includes the initial vortex with the core along $+z$. Center: intermediate state with the core along x. Right: final state with the core along $-z$.

cases, at 100 mT, the ratio equals 1, meaning the nanoparticle is fully saturated (see blue-framed snapshots in Figure S4(**G**)). A drop from this value marks vortex nucleation, which is, for the three configurations, with its core oriented along z, as indicated in the yellow-framed snapshots in Figure S4(**G**). The larger magnetostatic field in larger spheres counteracts the uniform state favoured by the Zeeman term, as validated by figures S4(**C**)-(**E**). As a result, the vortex is stabilized at stronger external field for the larger NS (from $\cong$ 40mT for $d = 150$ nm, to 25mT for $d = 110$ nm, and 12mT for $d = 100$ nm). In every case the core volume shrinks as the field decreases.
Still on Figure S4(**A**), it can be observed how, for the core-dominated reversal ($d = 100$ nm), the vortex never contracts below $V_{core}/V_{NS} = 1/3$. As a consequence, once the core flips, the in-plane moments gradually align with the field until full saturation is reached, and V_{core}/V_{NS} returns to 1. This gradual alignment of the in-plane moments once the core reverses manifests as linear trend in both the V_{core}/V_{NS} and the E_{demag} magnitudes, expected as the external field is applying a torque into the flux-closure moments, which are 90° oriented with respect to it. As such, a linear dependence with the externally applied field H is expected.
The linearity is broken above $d \geq 108$ nm, where the two-step reversal mechanism appears. Following volumetric factors, it can be observed how first, the core steadily contracts, until it reaches $V_{core}/V_{NS} \approx 1/3$. Below such a value, the flux-closure moments become the dominant fraction of the nanosphere, and as such, the magnetization rotates to bring a larger volume in alignment with the field at the cost of the core lying perpendicular to both the field and easy axis. The E_{demag} term plotted in Figure S4(**D**) mimics the V_{core}/V_{NS} trend, and a curved-shape, instead of the linear trend of the $d = 100$ nm case, starts to emerge. As d grows, the cost of a perpendicular core decreases relative to the gain due to the alignment of the flux-closure moments with the field, notably because of the significant increase of E_{demag}, producing a smoother V_{core}/V_{NS} profile (gray curve, $d = 150$ nm). The non-linear feature corresponding to the dynamics dominated by the flux-closure moments (marked in purple in Figure S4(**E**)) i.e., orientation of the vortex-core perpendicular to z, becomes broader compared to the $d = 110$ nm case, as a consequence of the larger E_{demag}. This non-linear dependence with the field, also clearly seen in the E_{Zeeman} term.
The geometrical considerations of the V_{core}/V_{NS} determines the coercivity of the NS. Along with Figure 2 of the main text, we have analyzed the consequences of this ratio by studying the size-dependence of the polar angle θ between the net NS magnetization and the $+z$ field, for the three sizes studied in Figure S4(**B**).
For the core-dominated reversal ($d = 100$ nm), the vortex core remains along z until its anisotropy barrier is crossed. Then, the entire magnetization flips sharply. In polycrystalline nanoflowers, however, multiple easy axes and grain boundaries can pin the core during reversal, thus some configurations briefly align the net moment perpendicular to the field, as observed in Figure 2 of the main text.
When the vortex core occupies less than one-third of the particle volume ($V_{\text{core}} < 1/3$), the reversal is governed by the in-plane flux-closure moments. These moments encircle the core (initially along z) and carry no net magnetization because rotational symmetry gives equal volumes magnetized along $\pm x$ and $\pm y$. Once V_{core} drops below 1/3, the combined magnetic volumes in the $\pm x$ (or equivalently, $\pm y$) direction exceed the core volume. Since uniaxial anisotropy depends only on the orientation of the magnetization and not on the sign of its direction, rotating the entire vortex profile by 90° (so the core tilts into the plane perpendicular to the field), becomes energetically favourable at low fields. The net moment therefore lies in the plane around remanence, as illustrated in Figure S4(**A**). The reversal becomes more gradual than in the core-dominated regime because of the intermediate situation of the core perpendicular to z prior to its switching to $-z$, being the flux-closure moments acting like an inertial body slowing the flipping down. This becomes even more evident for the larger NS ($d = 150$ nm). To highlight the difference between both situations, we have zoomed-in the region around remanence in the right inset. Note how for $d = 110$ nm, the polar angle is close to 90° at remanence (marked with a dashed red line), meaning the net magnetization is in-plane, and thus, yielding a 0 coercivity. The net magnetization for $d = 100$ nm stays however along the z direction until $\mu_0 H_z \cong -8.4$ mT, above its coercive field (see Figure S4(**D**)), and thus, the magnetization switches to the $-z$ direction.
Finally, Figure S4(**G**) captures representative magnetization snapshots for $d = 110$ nm to illustrate

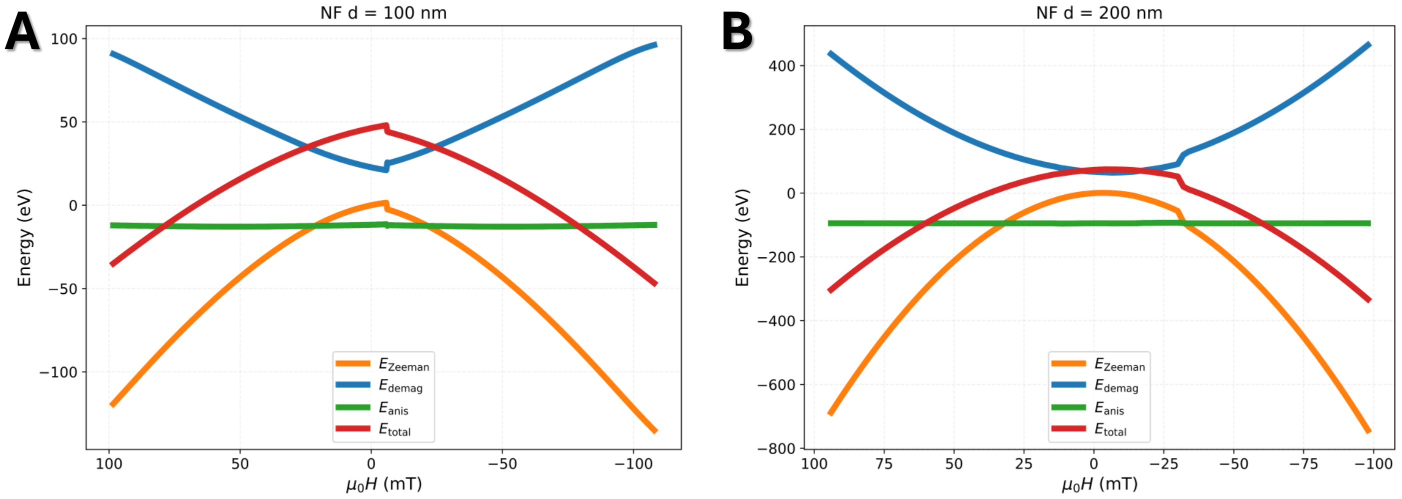

Figure S5: Zeeman E_{Zeeman}, demagnetizing E_{demag}, anisotropy E_{anis} and total E_{total} energy terms for $d = 100$ nm **(A)** and $d = 200$ nm **(B)** NFs with $k = 0.25$ and grain size of 15 nm as a function of the applied field $\mu_0 H_z$ for the magnetization reversal of the NFs.

the full rotation process. The top row shows the $y = 0$ slice, with the color representing the magnetization direction, while the bottom row emphasizes the vortex core in red. From left to right, the fields are $\mu_0 H_z = -100,\ 2.0,\ 0,\ -11.8$ and 100 mT. The vortex core rotates smoothly out of the z axis, passing through an intermediate state (middle frame at $\mu_0 H_z = 0$ mT) that does not appear in the smaller particle.

S6 Energy terms in nanoflowers

Figure S5 includes the Zeeman E_{Zeeman}, demagnetizing E_{demag}, anisotropy E_{anis} and total E_{total} energy terms for $d = 100$ nm **(A)** and $d = 200$ nm **(B)** NFs. As it can be observed, the anisotropy term is significantly smaller compared to the demagnetizing and Zeeman terms, consequence of the grain-texture of the NFs. As such, the total anisotropy is averaged out of the total amount of constituent grains, remaining like a "background" term to the total energy, which is dominated by the balance between demagnetizing and Zeeman energy terms.

S7 Balance between grain-boundary pinning and vortex coherence

In Section 3 of the main text, we showed that coercivity arises from a balance between grain-boundary pinning and the coherence of the vortex profile. Here we illustrate the limiting case of the absence of grain-boundary pinning, but a random distribution of uniaxial anisotropy directions across grains.. Figure S6 reveals the resulting magnetization: despite the remaining dipolar coupling between the grains, the coherence of the vortex profile is completely lost and instead a clear multi-domain pattern in which each grain behaves as an independent single-domain particle with its own random easy axis, is found. Averaging over these randomly oriented K_u directions drives the coercivity to zero. As such, this configuration is unable to reproduce the correct behaviour of a $d = 100$ nm nanoflower (NF), both in terms of the microstructure (vortex at remanence) and coercivity (~ 15 mT [4]) This extreme example highlights the essential role of inter-grain coupling in creating the hyperthermia "sweet spot", where coercivity, and thus heating efficiency, is maximized.

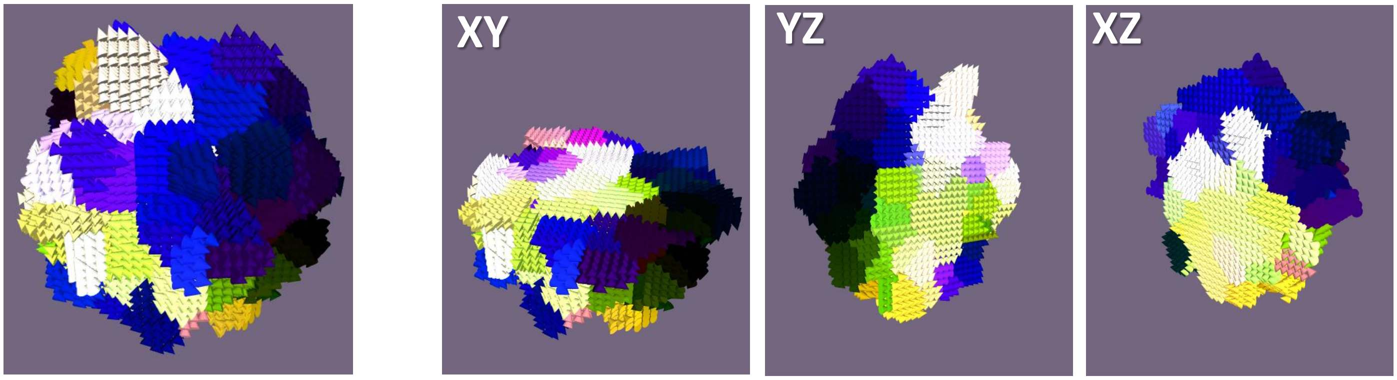

Figure S6: Remanent magnetization distributions, shown for a $d = 100$ nm NF with exchange uncoupled grains ($k = 0$), along with the corresponding three central planes XY, YZ and XZ. Each color represents a different magnetization direction, illustrating the uncoupled, multi-domain magnetization state.

References

[1] A. Vansteenkiste, J. Leliaert, M. Dvornik, M. Helsen, F. Garcia-Sanchez, B. Van Waeyenberge, *AIP Advances* **2014**, *4*, 10 107133.

[2] J. Leliaert, B. Van de Wiele, A. Vansteenkiste, L. Laurson, G. Durin, L. Dupré, B. Van Waeyenberge, *Journal of Applied Physics* **2014**, *115*, 23 233903.

[3] E. M. Jefremovas, L. Gandarias, I. Rodrigo, L. Marcano, C. Grüttner, J. Á. García, E. Garayo, I. Orue, A. García-Prieto, A. Muela, et al., *IEEE Access* **2021**, *9* 99552.

[4] C. Moya, M. Escoda-Torroella, J. Rodríguez-Álvarez, A. I. Figueroa, Í. García, I. B. Ferrer-Vidal, A. Gallo-Cordova, M. P. Morales, L. Aballe, A. F. Rodríguez, et al., *Nanoscale* **2024**, *16*, 4 1942.

[5] P. Bender, J. Fock, C. Frandsen, M. F. Hansen, C. Balceris, F. Ludwig, O. Posth, E. Wetterskog, L. K. Bogart, P. Southern, et al., *The Journal of Physical Chemistry C* **2018**, *122*, 5 3068.

[6] L. Storozhuk, M. O. Besenhard, S. Mourdikoudis, A. P. LaGrow, M. R. Lees, L. D. Tung, A. Gavriilidis, N. T. K. Thanh, *ACS Applied Materials & Interfaces* **2021**, *13*, 38 45870.

[7] H. Gavilán, E. H. Sánchez, M. E. Brollo, L. Asín, K. K. Moerner, C. Frandsen, F. J. Lázaro, C. J. Serna, S. Veintemillas-Verdaguer, M. P. Morales, et al., *ACS omega* **2017**, *2*, 10 7172.

[8] A. G. Roca, J. F. Marco, M. d. P. Morales, C. J. Serna, *The Journal of Physical Chemistry C* **2007**, *111*, 50 18577.

[9] H. Shokrollahi, *Journal of Magnetism and Magnetic Materials* **2017**, *426* 74.

[10] E. P. Sinaga, M. P. Adams, E. H. Hasdeo, A. Michels, *Physical Review B* **2024**, *110*, 5 054404.

[11] B. Gross, S. Philipp, E. Josten, J. Leliaert, E. Wetterskog, L. Bergström, M. Poggio, *Physical Review B* **2021**, *103*, 1 014402.

[12] J. Borchers, K. Krycka, B. Bosch-Santos, E. de Lima Correa, A. Sharma, H. Carlton, Y. Dang, M. Donahue, C. Grüttner, R. Ivkov, et al., *Small Structures* **2025**, *6*, 2 2400410.

[13] K. Pisane, S. Singh, M. Seehra, *Applied Physics Letters* **2017**, *110*, 22.

[14] J. A. R. Guivar, A. I. Martínez, A. O. Anaya, L. D. L. S. Valladares, L. L. Félix, A. B. Dominguez, *Advances in Nanoparticles* **2014**, *2014*.

[15] M. Bejko, Y. A. Yaman, A. Keyes, A. Bagur, P. Rosa, M. Gayot, F. Weill, S. Mornet, O. Sandre, *ChemPhysChem* **2024**, *25*, 22 e202400023.

[16] D. Sakellari, K. Brintakis, A. Kostopoulou, E. Myrovali, K. Simeonidis, A. Lappas, M. Angelakeris, *Materials Science and Engineering: C* **2016**, *58* 187.

[17] D. Serantes, D. Baldomir, *Nanomaterials* **2021**, *11*, 11 2786.

[18] U. Engelmann, E. M. Buhl, M. Baumann, T. Schmitz-Rode, I. Slabu, *Current Directions in Biomedical Engineering* **2017**, *3*, 2 457.

[19] M. Coïsson, G. Barrera, F. Celegato, L. Martino, S. N. Kane, S. Raghuvanshi, F. Vinai, P. Tiberto, *Biochimica et Biophysica Acta (BBA)-General Subjects* **2017**, *1861*, 6 1545.

[20] K. Simeonidis, C. Martinez-Boubeta, D. Serantes, S. Ruta, O. Chubykalo-Fesenko, R. Chantrell, J. Oró-Solé, L. Balcells, A. Kamzin, R. Nazipov, et al., *ACS applied nano materials* **2020**, *3*, 5 4465.

[21] E. Myrovali, K. Papadopoulos, G. Charalampous, P. Kesapidou, G. Vourlias, T. Kehagias, M. Angelakeris, U. Wiedwald, *ACS omega* **2023**, *8*, 14 12955.

[22] X. Liu, Y. Zhang, Y. Wang, W. Zhu, G. Li, X. Ma, Y. Zhang, S. Chen, S. Tiwari, K. Shi, et al., *Theranostics* **2020**, *10*, 8 3793.

[23] S. J. Holt, A. Petrocchi, M. Lang, S. A. Pathak, H. Fangohr, *arXiv preprint arXiv:2412.10466* **2024**.

[24] M. E. Schabes, H. N. Bertram, *Journal of Applied Physics* **1988**, *64*, 3 1347.

[25] R. Hertel, H. Kronmüller, *Journal of magnetism and magnetic materials* **2002**, *238*, 2-3 185.

[26] W. Rave, K. Fabian, A. Hubert, *Journal of Magnetism and Magnetic Materials* **1998**, *190*, 3 332.

[27] A. R. Muxworthy, W. Williams, A. P. Roberts, M. Winklhofer, L. Chang, M. Posfai, *Geochemistry, Geophysics, Geosystems* **2013**, *14*, 12 2430.

[28] D. Betto, J. Coey, *Journal of Applied Physics* **2014**, *115*, 17.

[29] W. Gan, M. Chandra Sekhar, D. Wong, I. Purnama, S. Chiam, L. Wong, W. Lew, *Applied Physics Letters* **2014**, *105*, 15.

[30] A. Kákay, L. Varga, *Journal of applied physics* **2005**, *97*, 8.

[31] A. Witt, K. Fabian, U. Bleil, *Earth and Planetary Science Letters* **2005**, *233*, 3-4 311.
